# Germline pathogenic variants detected by GenMineTOP: insight from a nationwide tumor/normal paired comprehensive genomic profiling test, in Japan

**DOI:** 10.1038/s10038-025-01389-z

**Published:** 2025-09-09

**Authors:** Eri Habano, Miho Ogawa, Kousuke Watanabe, Nana Akiyama, Hyangri Chang, Mirei Ka, Aya Shinozaki-Ushiku, Masahiko Tanabe, Masakazu Akahori, Toshimitsu Ichijo, Shuichi Tsutsumi, Kenji Tatsuno, Hiroyuki Aburatani, Hidenori Kage, Katsutoshi Oda

**Affiliations:** 1 Division of Integrative Genomics, Graduate School of Medicine, The University of Tokyo, Tokyo, Japan; 2 Next-Generation Precision Medicine Development Laboratory, Graduate School of Medicine, The University of Tokyo, Tokyo, Japan; 3 Department of Clinical Genomics, The University of Tokyo Hospital, Tokyo, Japan; 4 Department of Breast and Endocrine Surgery, The University of Tokyo Hospital, Tokyo, Japan; 5 KONICAMINOLTA INC., Tokyo, Japan; 6 Research Center for Advanced Science and Technology, The University of Tokyo, Tokyo, Japan; 7 Department of Respiratory Medicine, Graduate School of Medicine, The University of Tokyo, Tokyo, Japan

**Keywords:** Cancer genomics, Genetic testing

## Abstract

Comprehensive genomic profiling (CGP) expands treatment options for solid tumor patients and identifies hereditary cancers. However, in Japan, confirmatory tests have been conducted in only 31.6% of patients with presumed germline pathogenic variants (GPVs) detected through tumor-only testing. Paired tumor-normal analysis enables differentiation between somatic and germline variants. GenMineTOP, covered by Japan’s national health insurance since August 2023, analyzes paired samples and reports GPVs in 40 genes. This study provides an initial characterization of GPVs based on clinical findings collected during the first year of GenMineTOP implementation. We analyzed 1356 solid tumor patients who underwent GenMineTOP testing in the Center for Cancer Genomics and Advanced Therapeutics (C-CAT) database between August 2023 and July 2024, focusing on GPV detection rates, gene distribution, and comparisons with other CGP tests. Among the analyzed cancer types, GenMineTOP had a higher proportion of CNS/brain, soft tissue, bone, and head and neck cancers compared to other CGP tests. GPVs were detected in 73 patients (5.4%), with 38.2% classified as off-tumor. HR-related GPVs (*ATM*, *BRCA1*, *BRCA2*, *BRIP1*, *PALB2*, *RAD51C*, *RAD51D*) were found in both males (median age: 69) and females (median age: 54). Among males, 57.9% were aged 65 or older. GPVs may be detected in any cancer patients, including those with off-tumor findings, particularly in older male patients, especially in HR-related genes. These findings support the use of paired CGP to improve the diagnosis of hereditary cancers that could otherwise remain undetected.

## Introduction

Comprehensive genomic profiling (CGP) creates individual genomic profiles of tumors or tumor/normal tissues to explore therapeutic options. The primary objective of CGP testing is to guide patient treatment; however, it also has the potential to reveal the presence of germline pathogenic variants (GPVs). Here, GPVs are defined as germline-derived pathogenic variants in genes associated with autosomal dominant hereditary cancers. In Japan, based on the ACMG SF v3.2 [1], the Kosugi Group List designates 53 genes for the disclosure of secondary findings (Supplementary Table 1). This list includes genes deemed clinically actionable when GPVs are detected through CGP testing or other comprehensive genetic analyses.

In tumor-only panels, it is not possible to determine whether pathogenic variants, suspected to be of germline origin based on medical history, the Variant Allele Frequency (VAF) values and gene-specific germline conversion rates, are truly germline-derived. To confirm whether a presumed GPV is a true GPV, germline confirmatory testing (hereafter referred to as ‘confirmatory tests’) is necessary. In contrast, tumor/normal paired panels can detect GPVs without the need for confirmatory tests.

Reports from international studies indicate that the detection rate of GPVs through CGP ranges from 4.3% to 17.5% [2–8]. Identifying hereditary cancers not only benefits the affected individual but also facilitates health management for their relatives [3, 7, 9].

As of November 2024, five types of CGP have been clinically applicable in Japan. All genomic profiling data and clinical information are transferred to the Center for Cancer Genomics and Advanced Therapeutics (C-CAT) under written informed consent (consent rate: 99.7%), where the data is available for research purposes [10]. Notably, CGP testing under Japan’s public health insurance system is only applicable to patients who have completed or are expected to complete standard treatment. Consequently, this dataset consists predominantly of patients with poor-prognosis cancers. The five types of CGP tests include a tumor-only panel (FoundationOne^®^ CDx), two liquid biopsy panels (FoundationOne Liquid^®^ CDx and Guardant360^®^ CDx), and two tumor/normal paired panels (OncoGuide™ NCC Oncopanel System (NOP) [10, 11] and GenMineTOP). Among CGP tests in Japan, the most frequently used tumor-only panel is FoundationOne^®^ CDx (75%), while the liquid biopsy panels account for 11%. Tumor/normal paired panel usage remains limited to 14.3% in Japan [12, 13]. In tumor-only panels, patients undergoing CGP testing have completed standard treatment, and the rate of confirmatory tests for presumed GPVs is only 23.3–31.6% [14, 15]. In Japan, 9.6% of patients tested with CGP exhibited presumed GPVs, but only 49 patients (23.3%) underwent confirmatory tests, and among them, 15 patients (30.6%) were confirmed as GPVs. This suggests that hereditary cancers were diagnosed in only 0.6% of all patients who underwent FoundationOne^®^ CDx testing [14]. Therefore, hereditary cancers based on tumor-only panels are likely underdiagnosed.

GenMineTOP is the first CGP test in Japan that incorporates both DNA and RNA panels, covering 737 and 455 genes, respectively. Notably, the RNA panel is particularly advantageous for detecting a wide range of gene fusions, which can support both therapeutic decision-making and accurate diagnosis [16]. Until the approval of GenMineTOP in August 2023, NOP was the only tumor/normal paired panel available in Japan. Initially, NOP analyzed 114 genes, of which 16 were designated for GPV disclosure [17]. In February 2021, the number of target genes increased to 124, and all detected GPVs were subsequently disclosed. However, 20 genes the Kosugi Group List recommended were still not included [13]. In contrast, GenMineTOP analyzes 737 DNA panel genes, including all genes from the Kosugi Group List [18], with 40 highly recommended genes designated for reporting (Supplementary Fig. 1A). As of October 2024, the number of germline disclosure genes in GenMineTOP has been revised to 59 (Supplementary Fig. 1B), although tumor-normal paired testing is not a substitute for dedicated germline testing, which follows different pipelines and methodologies for germline analysis.

To date, nationwide real-world analysis on CGP-detected GPVs has only evaluated NOP (e.g., Yamaguchi et al., 2021). Furthermore, investigation of homologous recombination (HR) related genes other than *BRCA1/2* and the impact of differences in target genes between panels on GPV detection has been limited. The objective of this study was to clarify the current challenges in germline analysis as well as the clinical utility of GenMineTOP through its detection of GPVs.

## Materials and methods

### Patient samples of GenMineTOP from the C-CAT database

The data were obtained from the C-CAT database, curated by the National Cancer Center of Japan, which houses the CGP test results [10]. We accessed the C-CAT database to collect data on GenMineTOP cases registered between August 2023 and June 2024, downloading their clinical and genomic data on July 4, 2024. Additionally, we collected data on 21,169 CGP cases other than GenMineTOP, registered between July 2023 and June 2024, and downloaded their data on November 21, 2024. This study was approved by the Research Ethics Committee of the Faculty of Medicine at the University of Tokyo (approval number: 2021341G, G10114-(21)) and the C-CAT Data Utilization Review Board (approval number: CDU2022-026N).

### GenMineTOP testing

GenMineTOP, which analyzes 737 genes in its DNA panel and 455 genes in its RNA panel, has been covered by health insurance since August 2023 in Japan. It enables the detection of fusion genes in 455 genes and exon skipping in 5 genes with high sensitivity. In this study, GPVs were assessed for 40 hereditary cancer-related genes, although currently, GPVs for 59 genes are covered (Supplementary Fig. 1A, B). In GenMineTOP, germline single nucleotide variants (SNVs) and insertions/deletions (indels) in 40 genes are reported if classified as pathogenic or likely pathogenic in the ClinVar or Ambry Genetics Corporation databases. Additionally, for tumor suppressor genes, null variants (including nonsense mutations, splice site mutations, frameshift mutations, and start codon extensions) are also reported. All reported germline variants were manually reviewed to ensure accuracy. Allele-specific chromosomal copy number diagrams determined from over 8000 SNP probes are included in the Supplementary Report. VAF of GPVs is reported for both normal and corresponding tumor samples.

However, tumor VAF values were not available in the C-CAT database used in this study and thus could not be incorporated into the analysis.

### Germline pathogenic variants

Germline pathogenic variants (GPVs) were evaluated based on the 2015 guidelines of the American College of Medical Genetics and Genomics and the Association for Molecular Pathology (ACMG/AMP). Variants classified as pathogenic or likely pathogenic were defined as GPVs in this study. Variants associated with autosomal recessive inheritance were considered GPVs only when present in a homozygous or compound heterozygous state; heterozygous carriers of such variants were excluded. For tumor suppressor genes, null variants were also reported in the dataset. The system may capture variants with uncertain significance, such as C-terminal null variants (e.g., *BRCA2* p.R3384*); however, these variants were excluded from the definition of GPVs. For *POLE* variants, classification was based on recent recommendations specific to germline variants in the exonuclease domain (ED), emphasizing that pathogenic variants are typically non-disruptive missense mutations within the ED. In contrast, loss-of-function variants—such as nonsense or frameshift mutations—are generally not considered to be associated with cancer predisposition [19].

### Classification of tumors based on genetic alterations

Cancer susceptibility genes are linked to specific tumor types, termed “on-tumor”. In this study, the classification of on-tumor genes (Supplementary Table 2) was based on multiple sources, including the NCCN Clinical Practice Guidelines in Oncology (NCCN Guidelines^®^), such as Genetic/Familial High-Risk Assessment: Breast, Ovarian, Pancreatic, and Prostate (Version 3, 2025) and Colorectal, Endometrial, and Gastric (Version 4, 2024), among others [20–24]. For example, a pathogenic *BRCA1* variant is considered “on-tumor” in breast, pancreatic, ovarian, or prostate cancer because it increases the risk of these cancers. In contrast, the same variant is considered “off-tumor” in colorectal or liver cancer, as no clear risk association has been established.

### Definition of homologous recombination-related genes

*BRCA1* and *BRCA2* are well-established as key genes of the homologous recombination (HR) repair pathway. Recent studies have demonstrated that other HR-related genes, such as *RAD51C/D*, *PALB2*, *ATM*, *CHEK2*, *FANCA*, and *FANCD2*, also contribute to cancer susceptibility. Aberrations in these genes lead to impaired DNA damage repair, resulting in genomic instability and promoting cancer initiation and progression [25]. In this study, 15 genes (*BRCA1*, *BRCA2*, *ATM*, *BRIP1*, *BARD1*, *CDK12*, *CHEK1*, *CHEK2*, *FANCL*, *PALB2*, *PPP2R2A*, *RAD51B*, *RAD51C*, *RAD51D*, *RAD54L*) approved by the FDA for olaparib were defined as HR-related genes [26].

### Statistical analysis

Fisher’s exact test was used to compare patient backgrounds between GenMineTOP and other CGP tests, sex-based differences in GPV frequency, age group comparisons, and on-tumor/off-tumor detection rates among gene categories. The Wilcoxon rank-sum test was used to compare continuous variables such as median age. When multiple pairwise comparisons were performed, the Bonferroni correction was applied. A two-tailed *P*-value < 0.05 was considered statistically significant unless otherwise specified. All statistical analyses were conducted using JMP software (version 18.0.2, SAS Institute Inc., Cary, NC, USA).

## Results

### Comparison of patient backgrounds in GenMineTOP vs. other panels

**Fig. 1:**
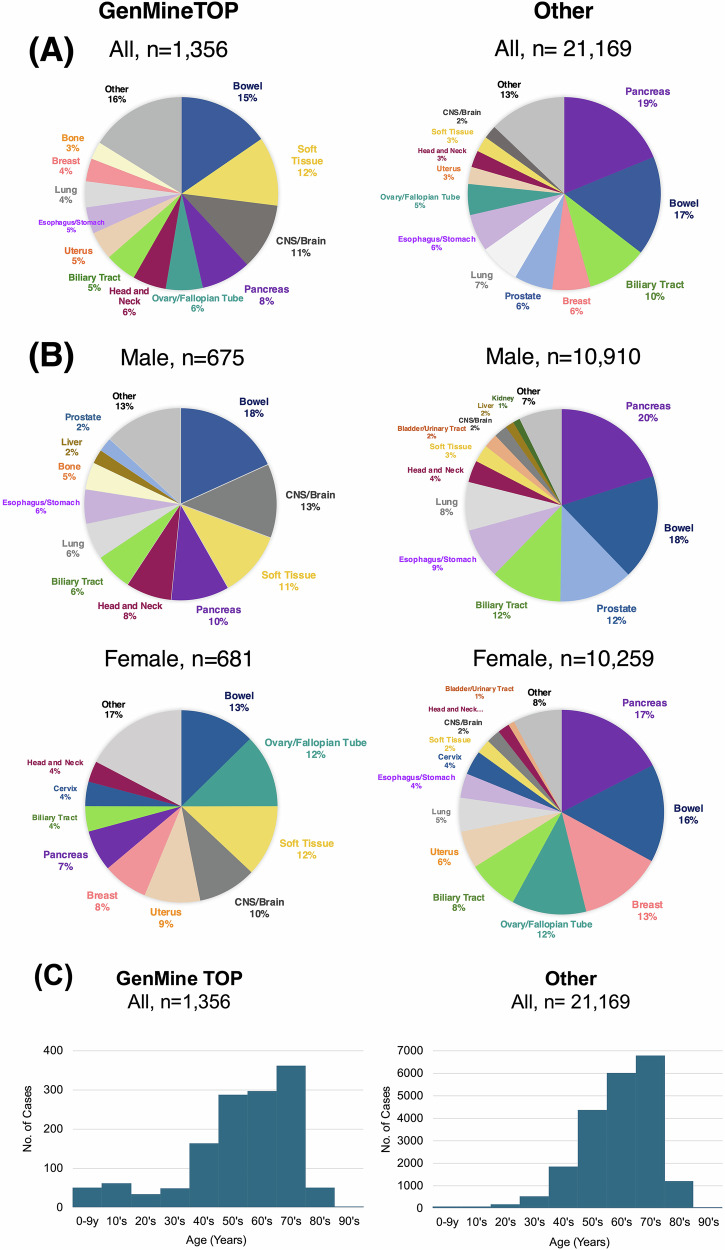
Comparison of patient backgrounds between GenMineTOP and other CGP cases. **A** Proportion of cancer types among all patients analyzed using GenMineTOP (*n* = 1356) and other CGP cases (*n* = 21,169). **B** Proportion of cancer types stratified by sex in GenMineTOP and other CGP cases. **C** Age distribution of all patients analyzed using GenMineTOP (*n* = 1356) and other CGP cases (*n* = 21,169)

This study included 1356 GenMineTOP cases from the C-CAT database. The median age was 61 (range: 0–90), with equal gender distribution (Supplementary Table 3). We compared 1356 GenMineTOP cases with 21,169 CGP cases excluding GenMineTOP cases (categorized as the “other” group) from the same period. In terms of cancer types analyzed, Fisher’s exact test identified significant differences in the proportions of specific cancer types

between the GenMineTOP and the other group (*p* < 0.05). GenMineTOP had a higher proportion of patients with CNS/brain (11% vs. 2%), soft tissue (12% vs. 3%), bone (3% vs. 0.4%), and head and neck (6% vs. 3%) cancers compared to the other group. Conversely, the proportions were lower for pancreatic (8% vs. 19%), and biliary tract (5% vs. 10%), lung (4% vs. 7%), esophagus/stomach (5% vs. 6%) cancers (Fig. 1A). When analyzed separately by gender, statistically significant differences were observed in males for CNS/brain (13% vs. 2%), soft tissue (11% vs. 3%), bone (5% vs. 0.4%), and head and neck (8% vs. 4%) cancers, where GenMineTOP had a higher proportion (*p* < 0.05, Fisher’s exact test). Conversely, significant differences were found in pancreatic (10% vs. 20%), prostate (2% vs. 12%), biliary tract (6% vs. 12%), esophagus/stomach (6% vs. 9%), and lung (6% vs. 8%) cancers, where the proportions were lower in GenMineTOP. In females, significant differences were observed for soft tissue (12% vs. 2%) and CNS/brain (10% vs. 2%), uterus (9% vs. 6%), head and neck (4% vs. 2%) cancers where GenMineTOP had a higher proportion (*p* < 0.05, Fisher’s exact test). However, GenMineTOP had significantly lower proportions in pancreas (7% vs. 17%), breast (8% vs. 13%), biliary tract (4% vs. 8%) cancers (Fig. 1B). Regarding age distribution, GenMineTOP had a significantly higher proportion of the pediatric, adolescent and young adult cancers (0–39 years old), compared to the other group (14.4% vs. 4.3%, *p* < 0.05, Fisher’s exact test) (Fig. 1C).

### Characteristics of patients with detected GPVs

**Fig. 2:**
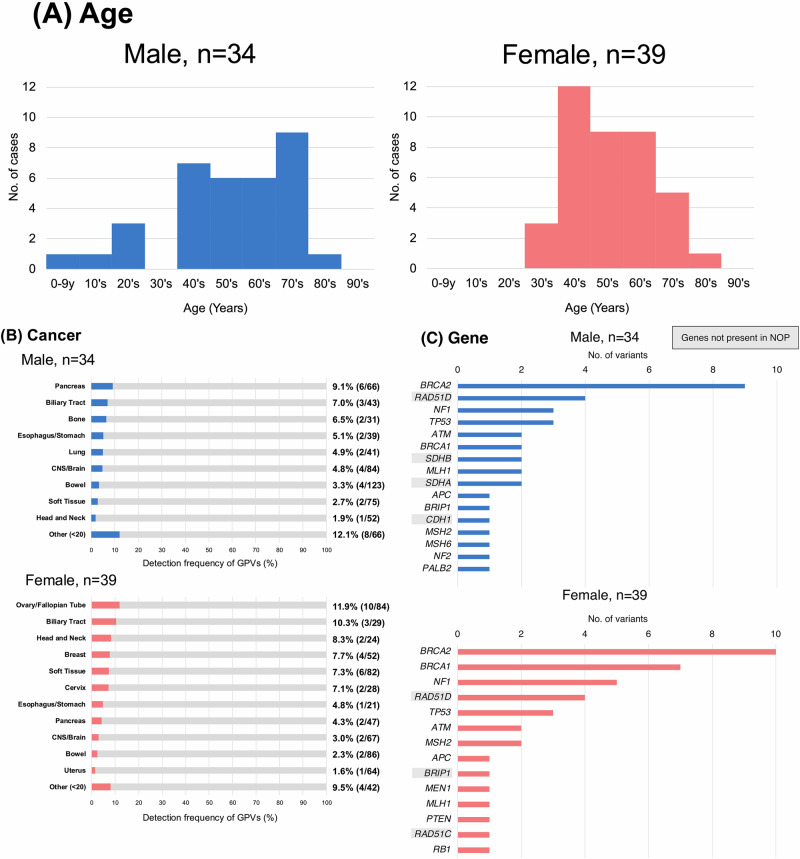
Characteristics of patients with detected GPVs. **A** Age distribution stratified by sex in patients with detected GPVs. **B** Detection frequency of GPVs by cancer type, stratified by sex. **C** Distribution of affected genes stratified by sex in patients with detected GPVs

As a result, 76 GPVs were detected in 73 out of 1356 patients (5.4%) (Supplementary Table 4). Among them, 34 patients (46.6%) were male, and 39 patients (53.4%) were female. The age distribution differed by gender. In males, GPVs were the most detected in their 70s (26.5%), whereas females were predominantly in their 40 s (30.7%). GPVs in individuals under 20 years old were exclusively detected in males at 14.7% (Fig. 2A). All 76 GPVs had a disclosure recommendation level of A or higher according to the Kosugi Group List. Among these, 19.7% (15 GPVs) were outside the scope of analysis by the NOP. The breakdown of these 15 GPVs was as follows: *RAD51D* (*n* = 8), *BRIP1* (*n* = 2), *SDHB* (*n* = 2), *SDHA* (*n* = 2), *CDH1* (*n* = 1) (Supplementary Table 5).

Cancers in which at least four GPVs were detected and the GPV detection rate was 3% or higher included ovary/fallopian tube (11.9%, 10/84), pancreas (7.1%, 8/113), soft tissue (5.1%, 8/157), biliary tract (8.3%, 6/72), CNS/brain (4.0%, 6/151), peripheral nervous system (20%, 4/20), and breast (7.7%, 4/52). The detection frequency of GPVs is shown for each cancer type, stratified by sex (Fig. 2B). Among females, the highest detection frequency was observed in ovary/fallopian tube (11.9%, 10/84), followed by biliary tract (10.3%, 3/29), and head and neck (8.3%, 2/24). Among males, the most frequent cancer type with GPVs was pancreas (9.1%, 6/66), followed by biliary tract (7.0%, 3/43), and bone (6.5%, 2/31). These results indicate that the distribution of GPVs varies depending on cancer type and sex (Fig. 2B).

Regarding genes, *BRCA2* was the most frequently detected gene in both males and females. Although *BRCA1* was more frequently observed in females, there was no statistically significant difference in the distribution of detected genes between sexes (Fig. 2C).

### Details of the detected GPVs (Age)

**Fig. 3:**
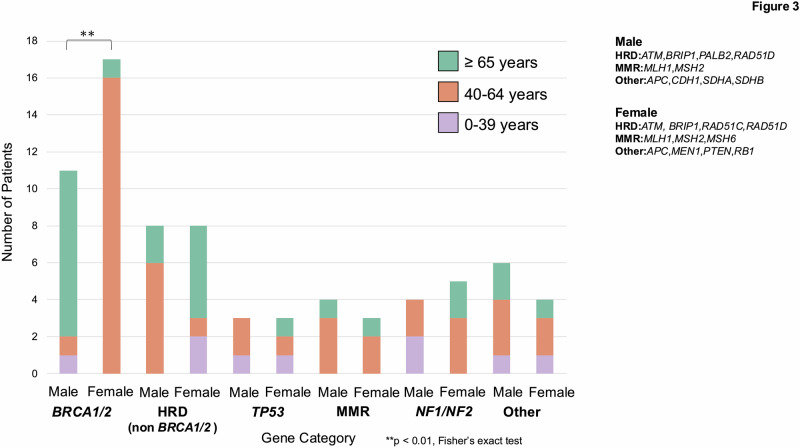
Age distribution by gene category and sex (stacked bar plot). Patients are grouped by gene category and stratified by sex (male, female). Bars represent the number of patients in three age groups: ≥65 years (green), 40–64 years (orange), and 0–39 years (purple). A significant difference in the proportion of elderly patients (≥65 years) between males and females in the *BRCA1/2* group was observed (*p* < 0.001, Fisher’s exact test). The specific genes included in each category for males and females are listed on the right

Patients were categorized by age (0–39, 40–64, ≥65 years). Statistical comparison was conducted between those aged ≥65 and <65. No overall sex-based difference was noted; however, in the *BRCA1*/*2* genes, males were significantly more likely to be aged ≥65 years (*p* < 0.01, Fisher’s exact test) (Fig. 3).

Among the eight patients (with nine variants) aged 0–39 years, *TP53* was the most frequently detected gene, identified in 2 out of 8 patients (25.0%). Other gene alterations included *APC*, *BRCA2*, *NF1*, *NF2*, *SDHB*, and *ATM/RAD51C*, each detected in one patient (12.5%, respectively). Due to the small sample size, statistical comparisons were not performed (Supplementary Fig. 2).

### Details of the detected GPVs (Cancer type: On-tumor/Off-tumor)

**Fig. 4:**
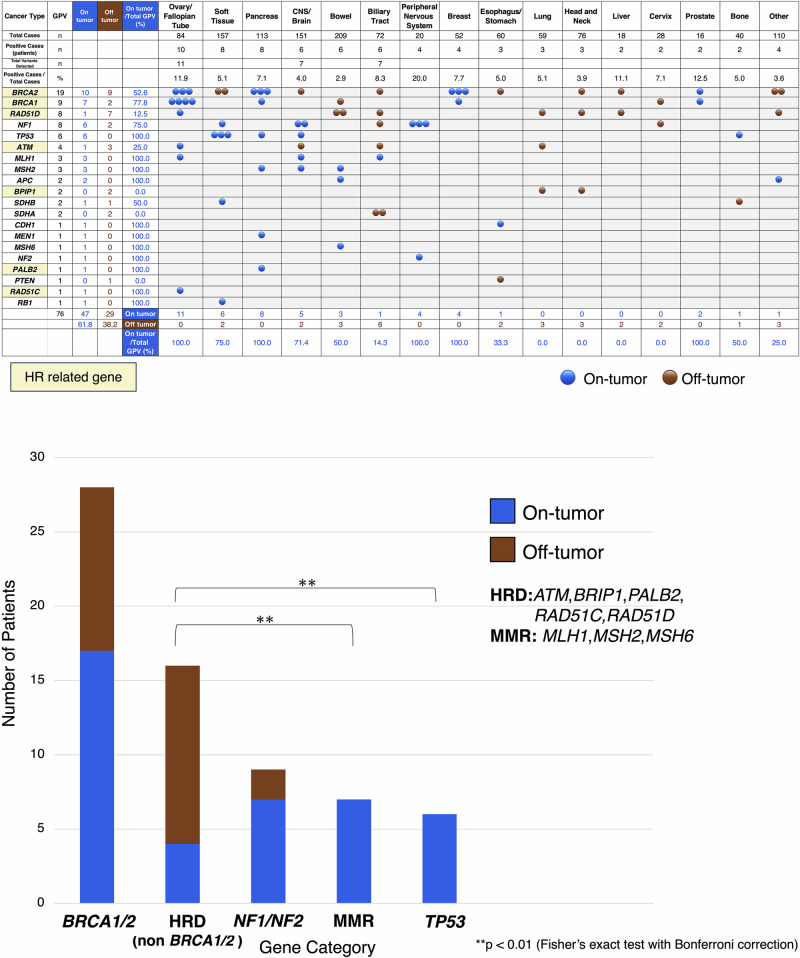
Details of detected GPVs by cancer type (On-tumor/Off-tumor). **A** A table with GPVs as row headers and cancer types as column headers, categorized into two groups: On-tumor (blue) and Off-tumor (brown). HR-related genes are highlighted in yellow. **B** On-tumor/Off-tumor distribution by gene category (stacked bar plot). Patients are categorized by gene group, showing the number of cases classified as on-tumor (blue) and off-tumor (brown)

The detected cancer types and genes are detailed in Fig. 4A. In the peripheral nervous system patients, the breakdown was *NF1* (*n* = 3) and *NF2* (*n* = 1). In the six cases of biliary tract cancer where GPVs were detected, variants in six different genes were identified. In the genes where GPVs were detected in at least three cases (eight genes: *BRCA1*, *BRCA2*, *RAD51D*, *NF1*, *TP53*, *ATM*, *MLH1*, *MSH2*), GPVs were identified in at least three different cancer types.

The detection rate of GPV for each cancer type was calculated. On-tumor findings accounted for 61.8% (47/76), while off-tumor findings comprised 38.2% (29/76). Among *BRCA1*/*2*, 60.7% (17/28) were classified as on-tumor. *BRCA1* was predominantly detected in ovarian cancer, with 77.8% (7/9) of patients classified as on-tumor. In contrast, *BRCA2* was detected across various cancer types with 52.6% (10/19) classified as on-tumor. The on-tumor detection rate was 77.8% (7/9) for *NF1*/*NF2*, 100% (6/6) for *TP53*, and 100% (7/7) for MMR genes, indicating that most of these variants were identified in clinically associated cancer types. In Fig. 4B, the on-tumor detection rate for the HRD-related gene group was significantly lower than that for both the TP53 group (*p* = 0.003) and the MMR group (*p* = 0.001), based on Fisher’s exact test with Bonferroni correction.

High tumor mutational burden (TMB-high; TMB-H) was observed in 8.2% (6/73) of cases. Five of these involved GPVs in mismatch repair (MMR) genes and showed TMB values ≥25 mut/Mb. The remaining case (TMB: 10.7 mut/Mb) did not involve MMR-GPVs. Overall, 71.4% (5/7) of patients with MMR-GPVs had TMB-H, suggesting a potential link to tumorigenesis. The other two MMR-GPV cases had low TMBs (2.7 and 4.8 mut/Mb), indicating limited functional impact (Supplementary Table 4).

### Gender and age of patients with GPVs in HR-related genes

**Fig. 5:**
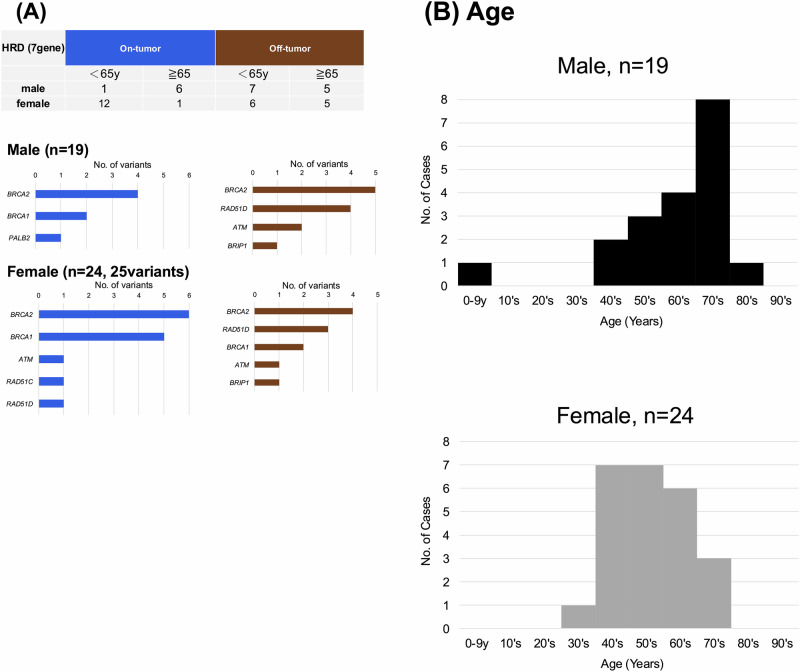
Gender and age distribution of patients with GPVs in HR-related genes. **A** Number of patients with GPVs in HR-related genes (7 genes), classified as on-tumor (blue) or off-tumor (brown). The number of patients for each detected HR-related gene is also presented. **B** Age distribution of patients with GPVs in HR-related gene, stratified by sex

GPVs were detected in seven HR-related genes: *BRCA2*, *BRCA1*, *RAD51D*, *ATM*, *BRIP1*, *PALB2*, and *RAD51C*. Among patients classified as on-tumor, only 14.3% (1/7) of males were aged ≤64 years, compared to 92.3% (12/13) of females (*p* < 0.001, Fisher’s exact test) (Fig. 5A). In contrast, among off-tumor patients, the proportion of those aged ≤64 years was similar between males (58.3%, 7/12) and females (54.5%, 6/11) (Fig. 5A). In the overall HRD-related group, the most common age group for males was the 70s (42.1%), while for females, it was the 40s–50s (58.4%). The median age was significantly higher in males (69.0 years, IQR: 56.0–75.0 years) than in females (54.5 years, IQR: 47.3–64.5 years) (Wilcoxon rank-sum test, *p* = 0.016) (Fig. 5B).

### Distribution of variant allele frequencies

**Fig. 6:**
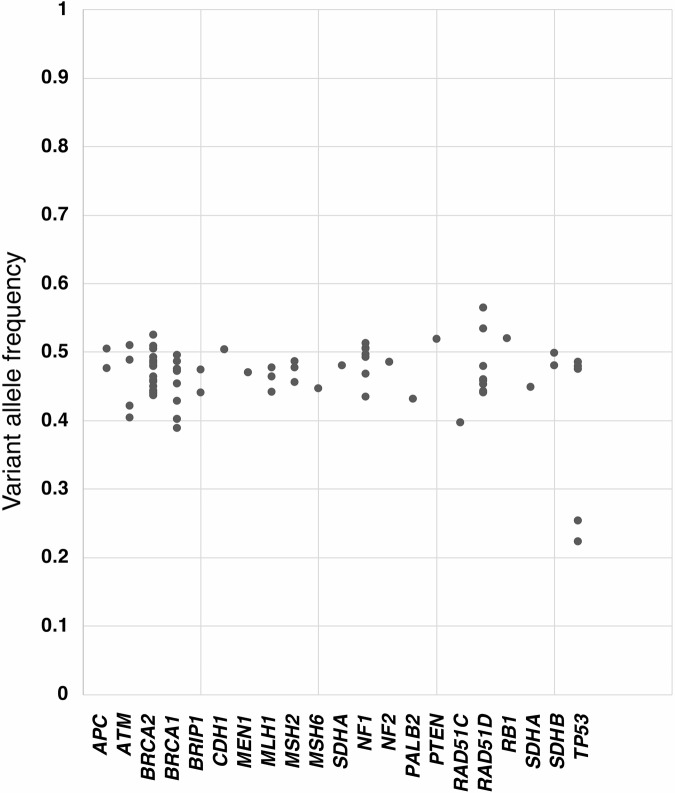
Distribution of variant allele frequencies (VAF) of GPVs in blood samples. The VAF values of the 76 detected GPVs are shown, with VAF represented on the vertical axis

The average VAF in blood samples analyzed by GenMineTOP was 0.47. Among the six *TP53* variants detected as GPV, two variants exhibited low VAF values. The patients with low VAF values included a 75-year-old female diagnosed with soft tissue sarcoma (*TP53* p.K132R, VAF = 0.224 [124/553]) and a 62-year-old male diagnosed with pancreatic cancer (*TP53* p.H179Y, VAF = 0.255 [116/455]). The cause of the low VAF in these patients cannot be clarified within this study and may possibly reflect clonal hematopoiesis of indeterminate potential (CHIP). Further germline-specific testing is required for a more comprehensive evaluation (Fig. 6).

### Detection and clinical implications of GPVs

Among the 73 patients in whom GPVs were detected, five were from our institution. Genetic counseling was conducted for four of these patients (Supplementary Table 6), including one patient whose GPV was already known prior to CGP testing. For the one patient who did not receive genetic counseling, the reason was a deterioration of the patient’s general condition, which prevented the disclosure of the CGP test results. In one patient (Patient 4) with a *BRCA2* GPV, a family history of breast cancer facilitated discussions regarding diagnostic evaluation for the patient’s relatives.

As allele-specific copy number graphs are available in “Supplementary Information” (Supplementary Fig. 3A), we assessed the presence of loss of heterozygosity (LOH) by integrating allele-specific copy number graphs (Supplementary Fig. 3B–E), and variant allele frequencies (VAFs) in tumor and normal samples. LOH was inferred when the copy number of the minor allele (shown in blue) was markedly decreased, indicating allelic imbalance.

Considering the histologically estimated tumor content ratio, we presumed that LOH at the locus of the GPV gene was coexistent with GPV in Patients 1, 2 and 5. In Patient 3, a somatic pathogenic variant coexisted with GPV in the *NF1* gene. However, in Patient 4 with a *BRCA2* GPV, neither LOH nor a somatic pathogenic variant was identified, suggesting that the *BRCA2* GPV may be unrelated to the liver cancer and instead represents an incidental finding.

## Discussion

This study demonstrated the utility of GenMineTOP in detecting GPVs across diverse cancer types and age groups. In this study, we addressed the expansion of target genes and the significance of the tumor-normal paired panel through analyses based on age, sex, and on/off tumor classification.

In the first year of insurance coverage, GenMineTOP was found to include a high proportion of CNS/brain, soft tissue, bone, and head and neck cancers. As previously noted, one of the distinguishing features of GenMineTOP is its ability to detect gene fusions through RNA-based analysis. This technical advantage may have contributed to its preferential selection for sarcomas and certain other tumor types in our cohort, as shown in Fig. 1A. Compared to CGP tests that analyze only DNA, the inclusion of RNA profiling may provide additional clinical value for tumors in which fusion genes are more prevalent [16]. Previous studies have reported a wide range of GPV detection rates through CGP testing, from 4.3% to 17.5% [2–8]. Such variation can be attributed to multiple factors, including the number of genes analyzed, variant classification criteria, patient demographics (e.g., cancer types), and ethnic background. Notably, a recent international study using tumor/normal paired CGP testing across multiple cancer types reported an overall GPV detection rate of 7.3%, with higher rates in ovarian (13.8%) and breast cancers (10.8%), and lower rates in bladder (6.6%) and lung cancers (5.8%) [27]. These differences underscore the importance of tumor type when interpreting GPV detection rates. In our analysis, differences in cancer type distribution between GenMineTOP and other CGP tests likely contributed to the variation in GPV detection, and should be considered when making cross-cohort comparisons.

Analysis by cancer type revealed that, in addition to ovarian, breast, and pancreatic cancers, which are widely known for their high frequency of hereditary cancers, GPVs were detected at a rate of 3% or higher, including in soft tissue, CNS/brain, biliary tract, and peripheral nervous system cancers. This indicates the need to be aware of GPVs across a diverse range of cancer types.

The GPV detection rate was 5.4%, while the detection rate in NOP was 4.1–4.5% (*n* = 3739–6313) [12, 13]. Notably, six genes with 15 unique variants were exclusively detected by GenMineTOP but not in NOP, with *RAD51D* accounting for eight of these variants. Genes in the RAD51 protein family play a crucial role in homologous recombination and DNA repair. *RAD51D* has been associated with an increased risk of ovarian cancer, with an estimated germline prevalence of approximately 1% in ovarian cancer patients [28–32]. Additionally, its association with breast cancer risk has been reported [33–36]. *BRIP1*, detected in two patients, has also been linked to an increased ovarian cancer risk [37–39]. Other detected genes included *SDHB*, *CDH1*, and *SDHA*, highlighting the potential for comprehensive germline testing to identify individuals who could otherwise remain undiagnosed. At the time of this analysis, GenMineTOP included 40 genes for germline variant reporting. However, as of October 2024, the gene panel has expanded to 59 genes, incorporating all genes recommended in the Kosugi Group List (Supplementary Fig. 1B). Further validation of newly added genes is warranted.

This study confirmed that GPVs, particularly in HR-related genes such as *BRCA2* and *RAD51D*, were also detected in older patients. This likely reflects the age distribution of cancers associated with HR-related genes. For instance, prostate cancer primarily affects older men, and pancreatic cancer is more common in older individuals, which may explain the higher detection rate of GPVs in these groups [40, 41]. GPV age distribution also varied by sex, with 92.3% of HR-related genes detected in younger women (<65 years), reflecting the early-onset nature of breast and ovarian cancer [42, 43].These findings underscore the importance of CGP testing across all age groups.

Of note, 38.2% of GPVs in this study were detected in off-tumor patients. While the role of these GPVs in tumorigenesis remains unclear, their detection suggests the potential for underlying hereditary cancers. The detection of *BRCA1/2* variants in off-tumor patients, for example, may inform cancer surveillance strategies for other related cancers and contribute to family health management. Particularly, 75.0% of non-*BRCA1/2* HR-related genes were detected in off-tumor patients, suggesting their possible but currently unestablished contribution to tumorigenesis. Momozawa et al., have reported that *BRCA1/2* variants may increase the risk of gastric, esophageal, and biliary tract cancers [44]. Additional data collection is necessary to clarify the role of non-*BRCA1/2* genes. However, GPV detection in off-tumor patients may also be incidental, necessitating consideration of the psychological and medical implications for patients and their families. These findings highlight the critical role of genetic counseling in managing unexpected results.

Two low VAF pathogenic *TP53* variants were detected, suggesting CHIP, somatic mosaicism, or germline mosaicism. However, as this study did not analyze normal tissues other than blood, distinguishing these possibilities was not feasible. Notably, the detected variants (p.K132R, p.H179Y) were not among previously reported CHIP hotspots (p.R248W, p.R273H, p.Y220C) [45], emphasizing the complexity of interpreting low-frequency variants in CGP data. It is important to emphasize that tumor-normal paired CGP testing is not a substitute for dedicated germline testing, which employs distinct analytical pipelines and confirmatory procedures tailored to germline variant interpretation. In particular, genes such as *TP53* are prone to somatic alterations in blood cells (e.g., CHIP), and low VAF variants in such genes require careful evaluation through additional confirmatory testing. These limitations should be carefully considered when interpreting germline variant findings from tumor-normal CGP data. and the need for additional confirmatory tests.

GenMineTOP allows estimation of LOH for each GPV through its allele-specific chromosomal copy number diagrams, in addition to VAF in both normal and tumor samples. This enables the estimation of whether the detected GPV is a driver of tumorigenesis or an incidental finding. However, in the present study, we used C-CAT-registered data, which did not include tumor VAF values or copy number figures. As a result, LOH could not be assessed in most cases, limiting our ability to evaluate the functional significance of each GPV. Notably, five cases included in the C-CAT data originated from our institution, where access to the full GenMineTOP reports allowed us to review copy number plots and VAF values. For these cases, LOH assessment was feasible and used to support interpretation of the GPVs.

GenMineTOP classifies variants based on established databases like ClinVar and Ambry Genetics Corporation databases, reporting pathogenic or likely pathogenic variants, including null variants in tumor suppressor genes. However, the system may capture variants with uncertain significance, such as C-terminal null variants, necessitating molecular tumor board review for refinement. Further standardization and functional studies could improve the accuracy of germline variant interpretation in precision oncology.

This study has several limitations. First, its retrospective design may introduce selection bias, as only patients undergoing CGP testing were included. Second, reliance on pre-existing databases (C-CAT) limits access to key clinical details such as family and treatment history, which may influence GPV interpretation. Additionally, genetic counseling and confirmatory test results were not always available for patients diagnosed outside our institution, making it unclear whether *BRCA1/2* variants detected through CGP had been previously identified. Furthermore, GenMineTOP is not a germline-focused genetic test and does not employ a germline-specific analytical pipeline, such as the detection of large deletions by MLPA, which fundamentally differentiates it from conventional hereditary cancer testing. Because no confirmatory test data was available to validate the pathogenicity of the detected GPVs, we cannot draw definitive conclusions regarding the analytical or clinical utility of these findings. Lastly, the impact of the expanded 59-gene panel (as of October 2024) on GPV detection and clinical outcomes requires further evaluation.

GenMineTOP demonstrated its utility as a CGP test capable of detecting clinically actionable GPVs across a wide range of cancer types and age groups through tumor/normal paired analysis. By incorporating the Kosugi Group List, GenMineTOP covers a broader range of genes and can identify variants undetectable by other panels, underscoring its potential role in advancing precision oncology.

## Supplementary information

Supplementary Figure 1 (A–B) Kosugi Group List Disclosure Genes and Target Genes in Each Panel

Supplementary Figure 2. Distribution of GPVs by Cancer Type and Age Group

Supplementary Figure 3 (A–E)

Supplementary Table 1. Disclosure Recommendation List of Germline Findings in Cancer Genomic Profiling Tests (Kosugi Group List ver.4.2)

Supplementary Table 2. Classification of On-Tumor and Off-Tumor Cancer Types by Gene

Supplementary Table 3. Clinical Characteristics of Patients Who Underwent Testing With the GenMineTOP

Supplementary Table 4. Details of 73 Patients with GPVs in GenMineTOP

Supplementary Table 5. Recommendation Grades of GPVs Detected in GenMineTOP Based on the Kosugi Group List

Supplementary Table 6. Details of GPVs and Genetic Counseling for 5 Patients in Our Institution

Tables Legend

coi disclosure for corresponding author
