# Supplementary material for: Germline pathogenic variants detected by GenMineTOP: insight from a nationwide tumor/normal paired comprehensive genomic profiling test, in Japan
Source: J Hum Genet. 2025 Sep 9;71(1):1–11. doi: 10.1038/s10038-025-01389-z (PMC12689426; doi:10.1038/s10038-025-01389-z)

Supplementary Figure 1A. Kosugi Group List Disclosure Genes and Target Genes in Each Panel

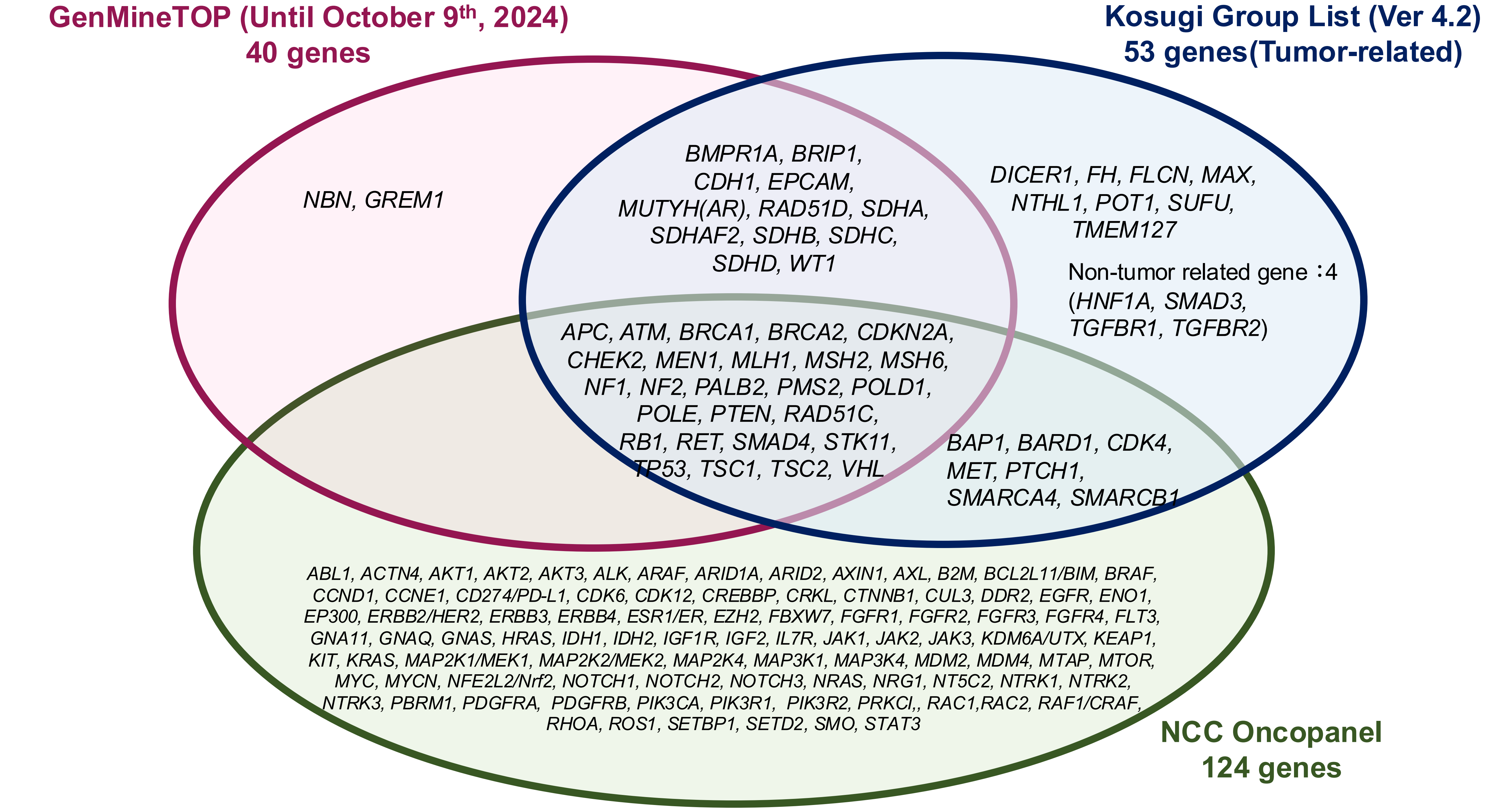

Supplementary Figure 1B. Kosugi Group List Disclosure Genes and Target Genes in Each Panel

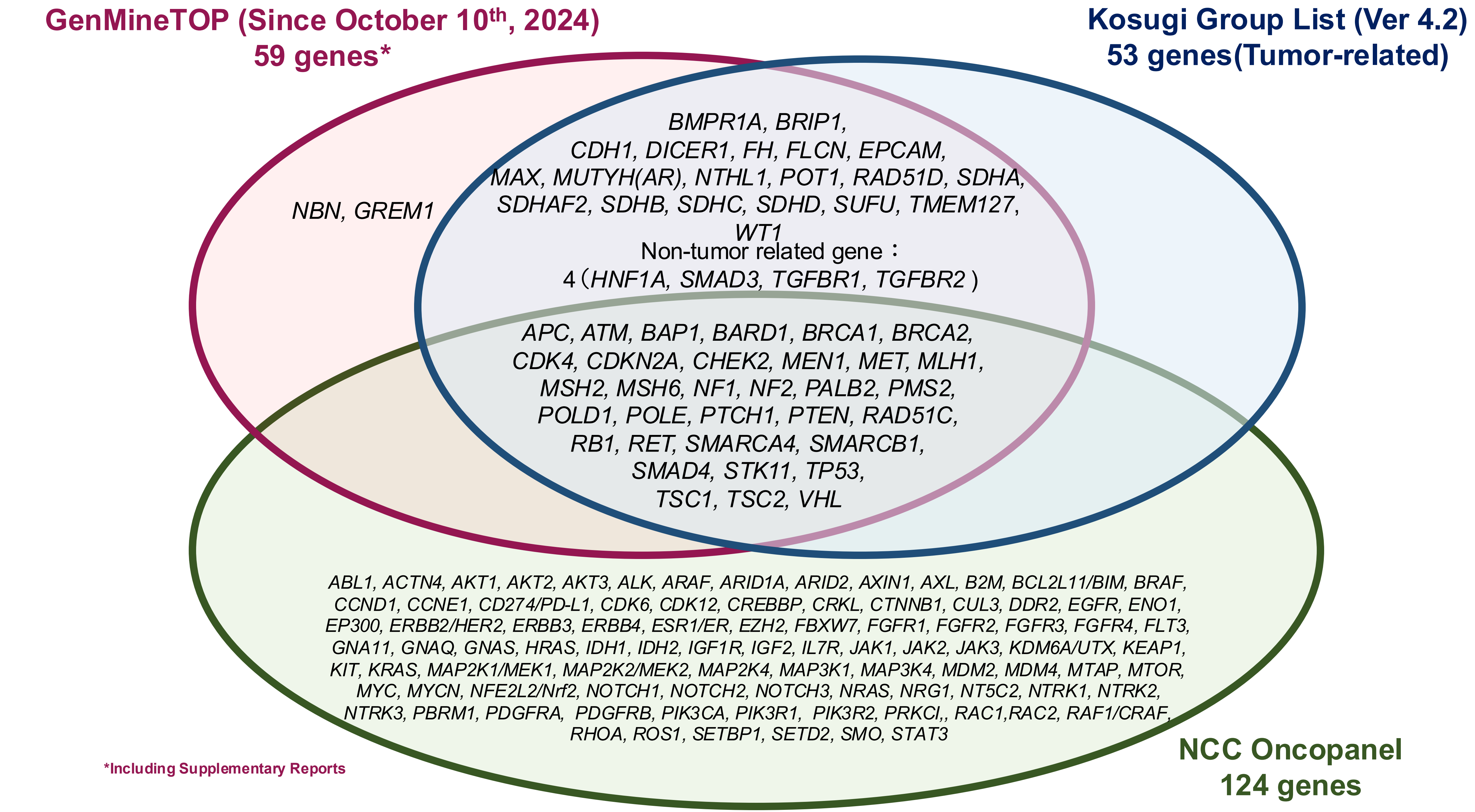

Supplement: Supplementary file 1 — Supplementary Figure 1 (A–B) Kosugi Group List Disclosure Genes and Target Genes in Each Panel [file 10038_2025_1389_MOESM1_ESM.pdf]
