# Supplementary figures and images for: Germline pathogenic variants detected by GenMineTOP: insight from a nationwide tumor/normal paired comprehensive genomic profiling test, in Japan
Source: J Hum Genet. 2025 Sep 9;71(1):1–11. doi: 10.1038/s10038-025-01389-z (PMC12689426; doi:10.1038/s10038-025-01389-z)

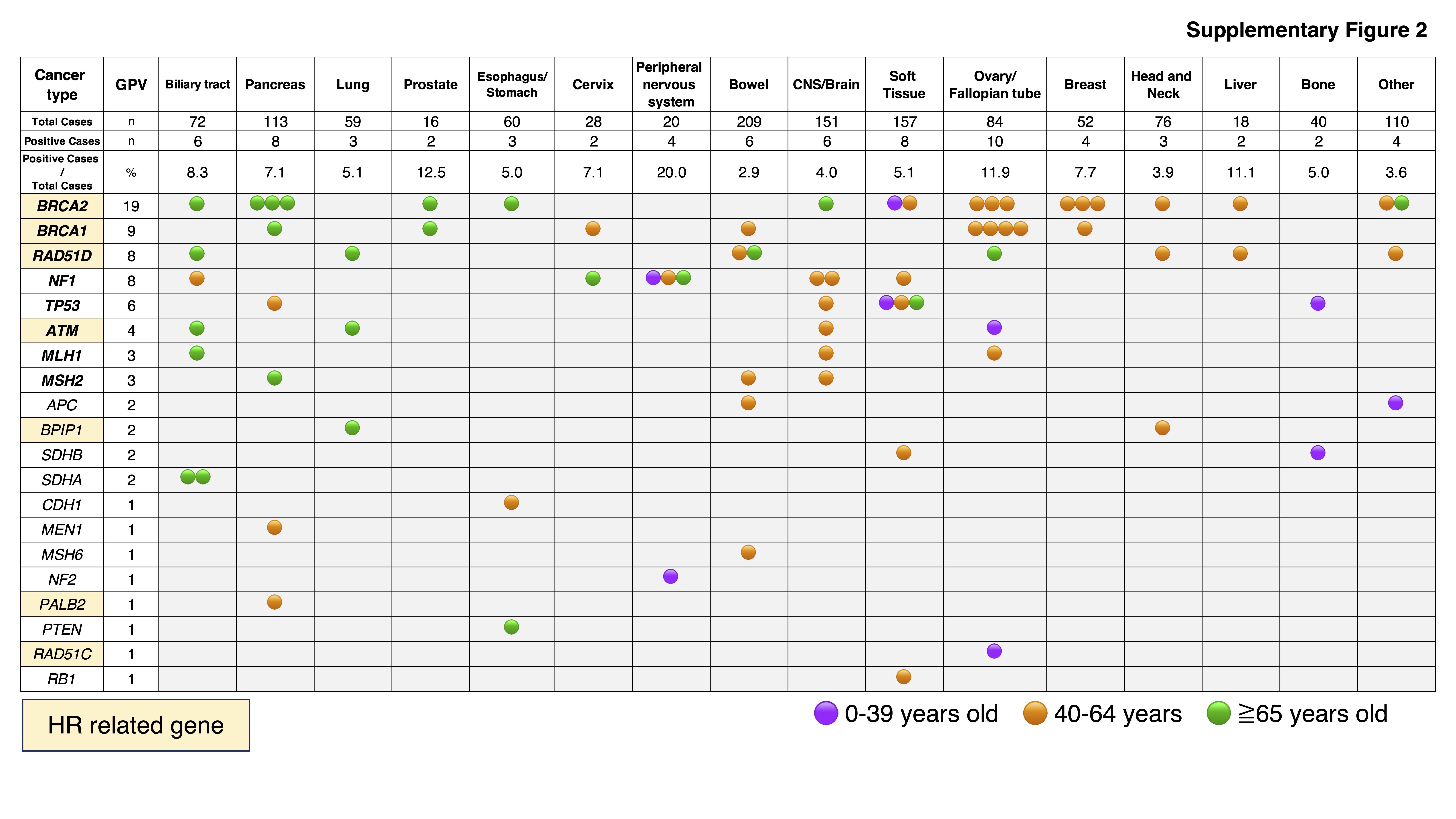

Supplement: Supplementary file 2 — Supplementary Figure 2. Distribution of GPVs by Cancer Type and Age Group [file 10038_2025_1389_MOESM2_ESM.jpg]

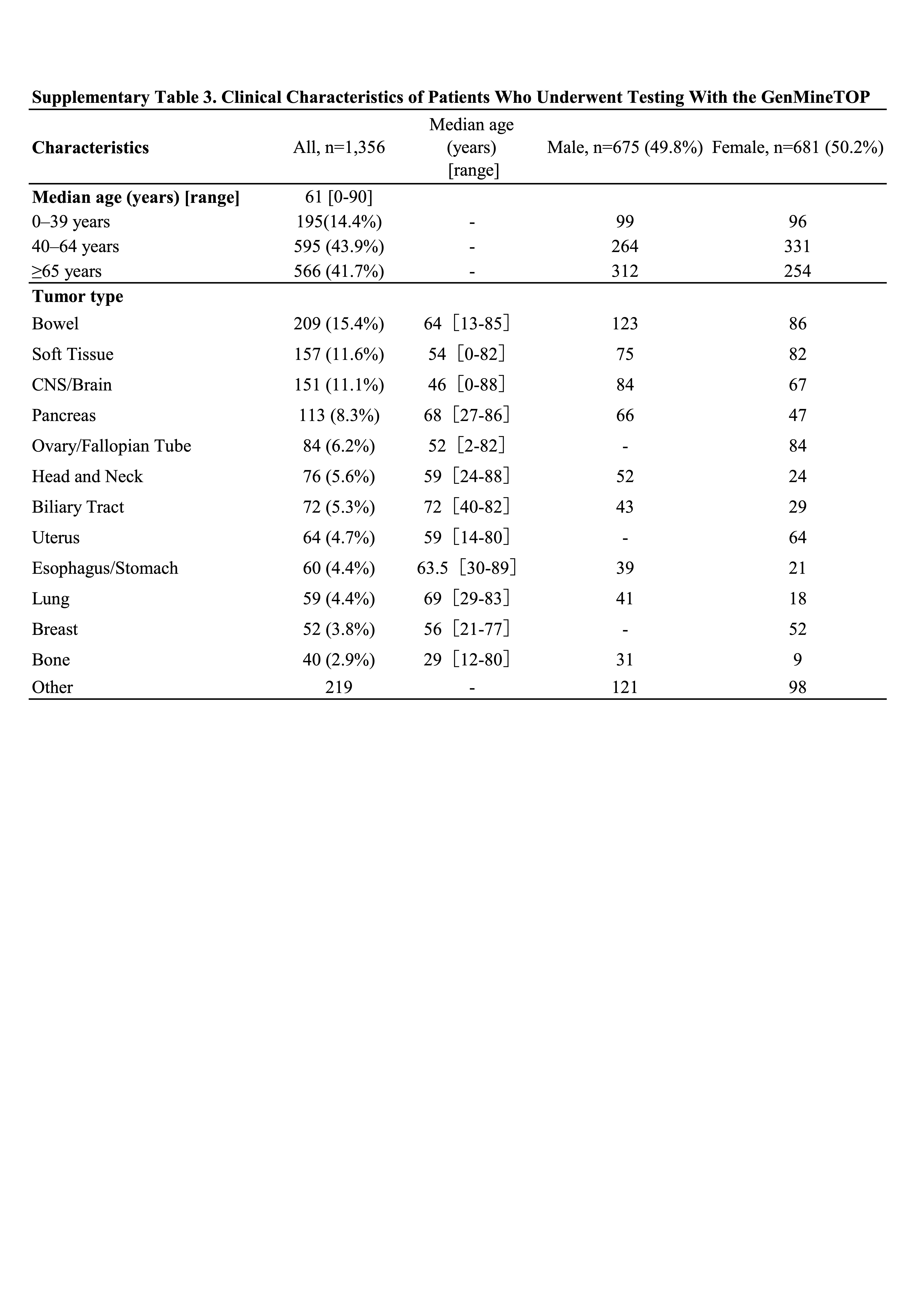

Supplement: Supplementary file 6 — Supplementary Table 3. Clinical Characteristics of Patients Who Underwent Testing With the GenMineTOP [file 10038_2025_1389_MOESM6_ESM.jpg]

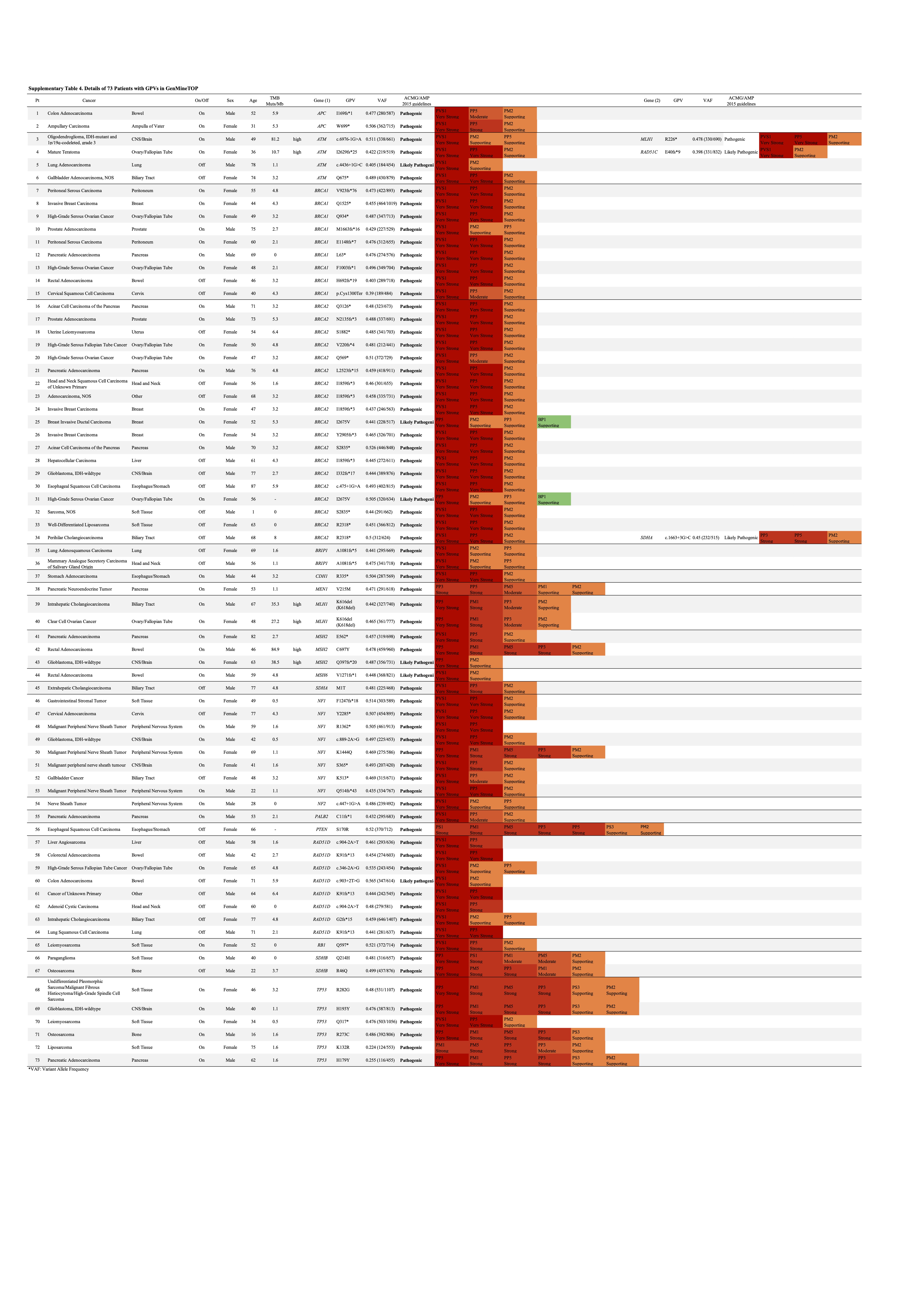

Supplement: Supplementary file 7 — Supplementary Table 4. Details of 73 Patients with GPVs in GenMineTOP [file 10038_2025_1389_MOESM7_ESM.jpg]

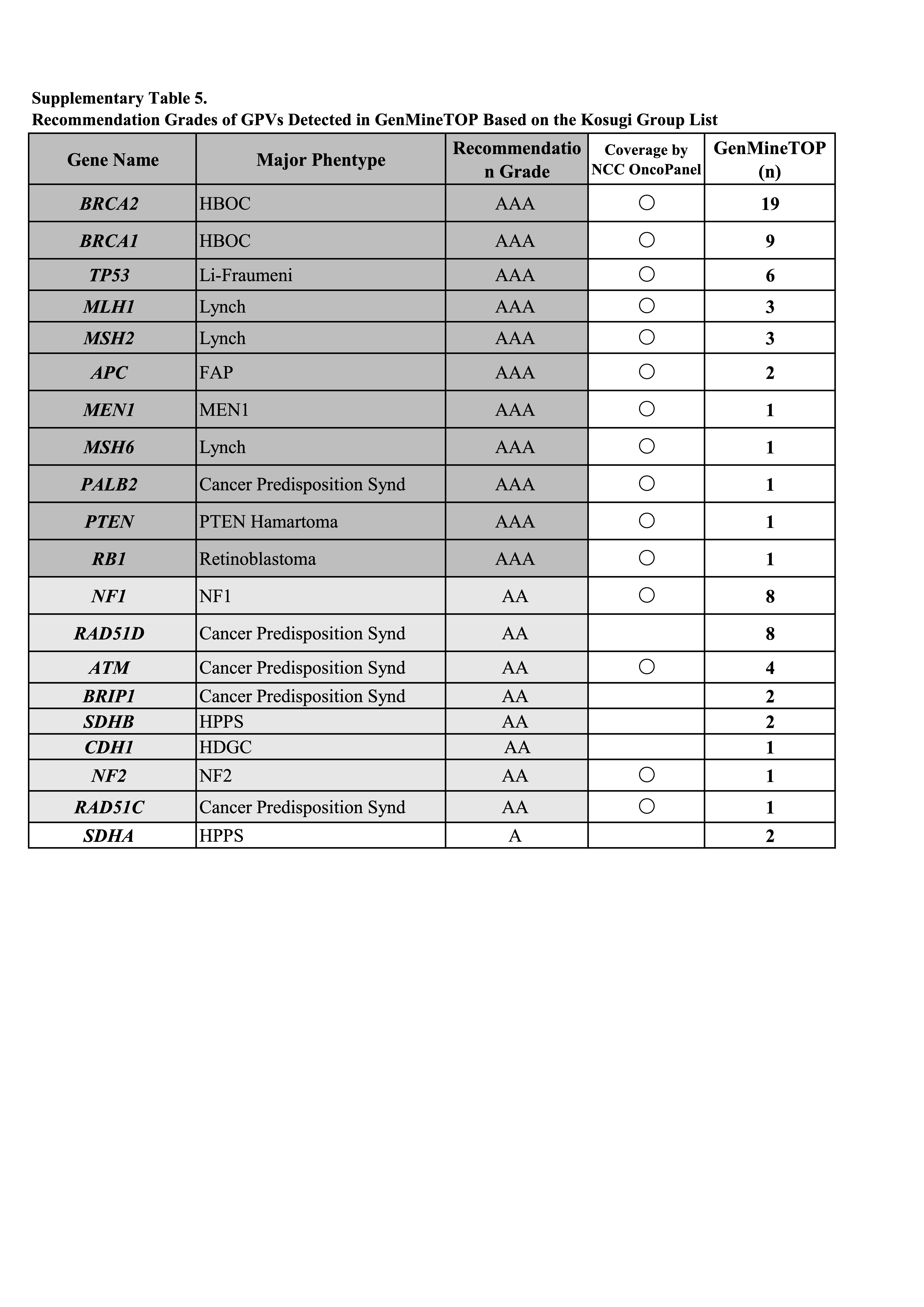

Supplement: Supplementary file 8 — Supplementary Table 5. Recommendation Grades of GPVs Detected in GenMineTOP Based on the Kosugi Group List [file 10038_2025_1389_MOESM8_ESM.jpg]

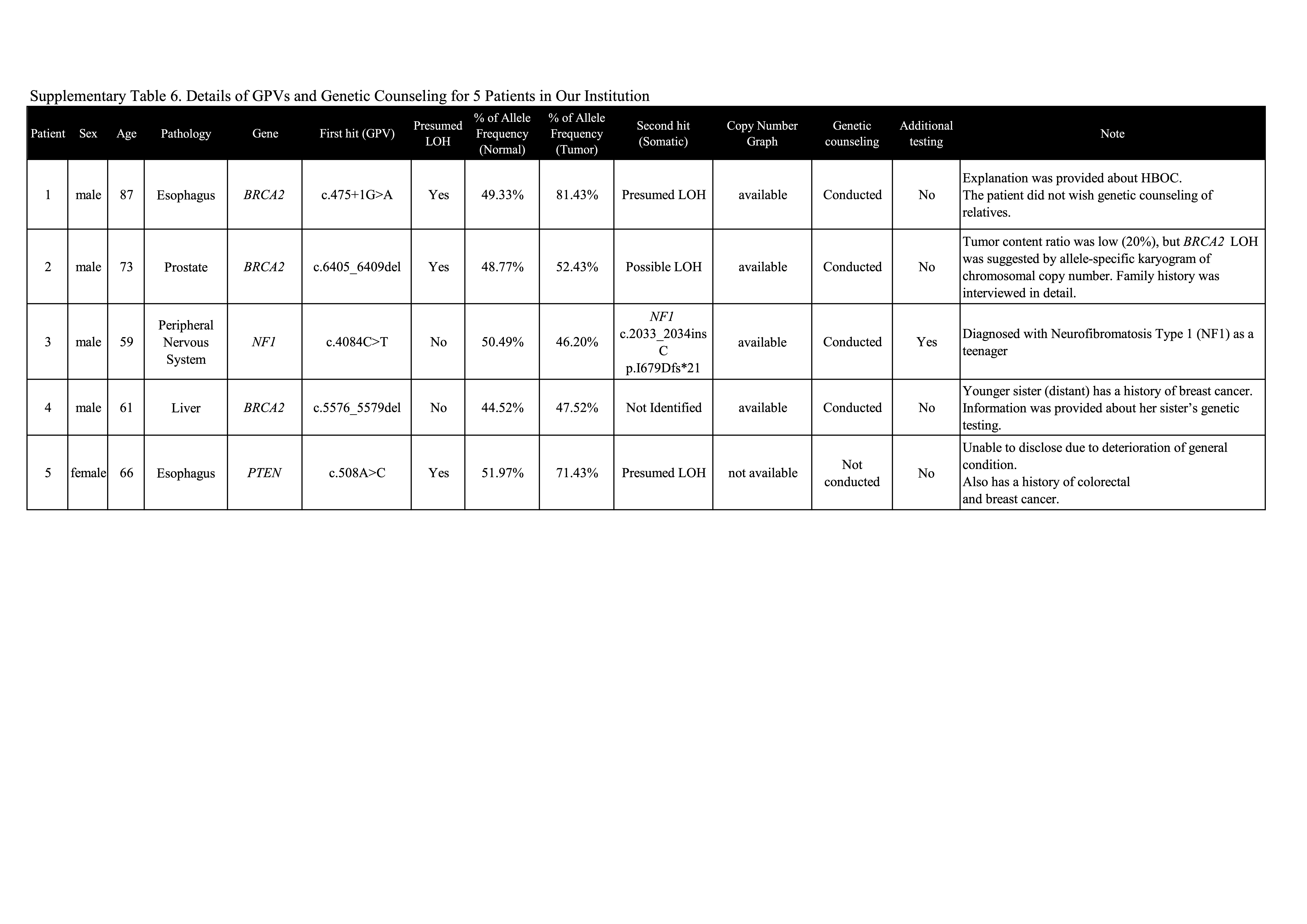

Supplement: Supplementary file 9 — Supplementary Table 6. Details of GPVs and Genetic Counseling for 5 Patients in Our Institution [file 10038_2025_1389_MOESM9_ESM.jpg]
