# Supplementary material for: Germline pathogenic variants detected by GenMineTOP: insight from a nationwide tumor/normal paired comprehensive genomic profiling test, in Japan
Source: J Hum Genet. 2025 Sep 9;71(1):1–11. doi: 10.1038/s10038-025-01389-z (PMC12689426; doi:10.1038/s10038-025-01389-z)
Supplement: Supplementary file 3 — Supplementary Figure 3 (A–E) [file 10038_2025_1389_MOESM3_ESM.pdf]

How to Interpret Copy Number and Allele-Specific Copy Number Graphs

Supplementary Figure 3A

Supplementary Information

1 Copy number graph

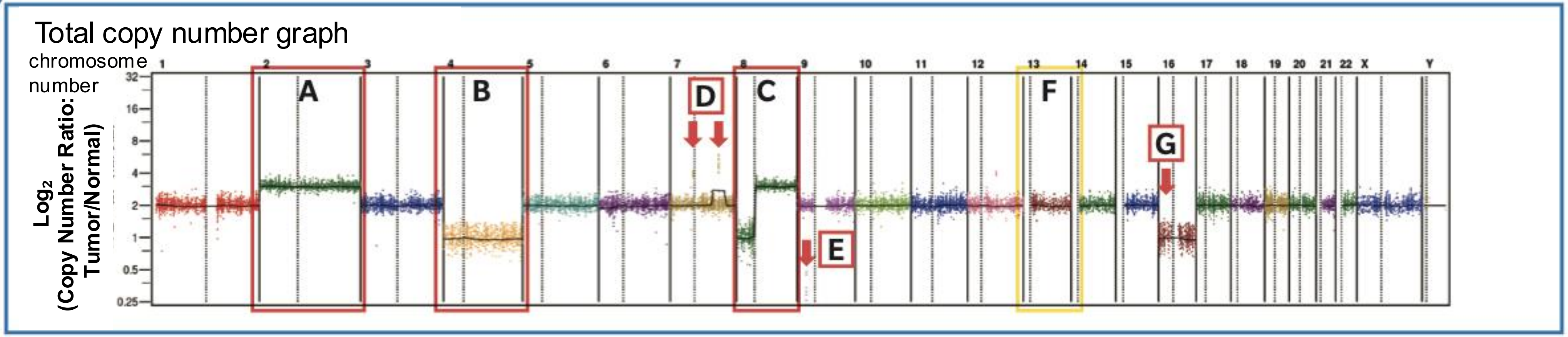

Chromosome

2 Copy number graph by allele

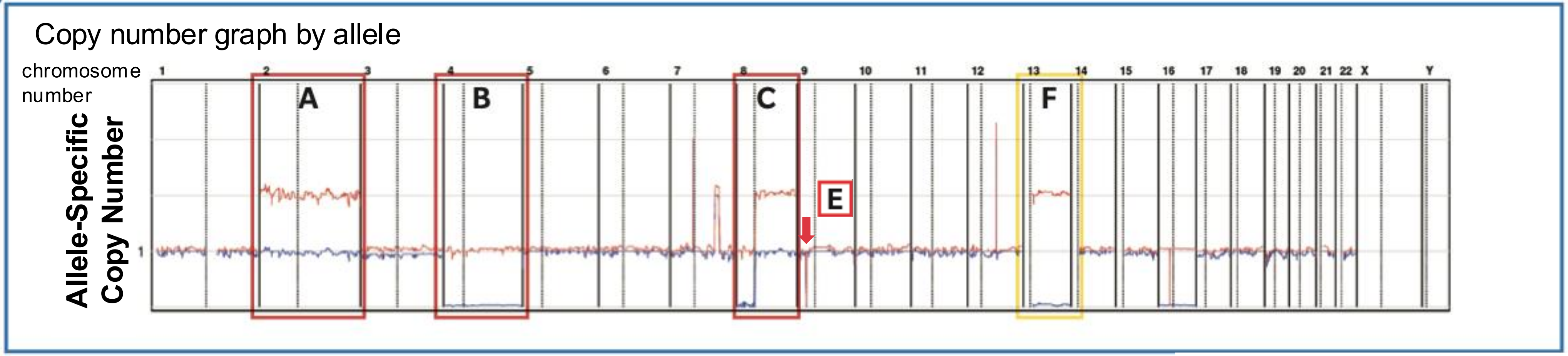

A: Duplication
B: Loss of heterozygosity (LOH)
C: Arm-level gain or loss
D: Gene-level gain

E: Homozygous deletion
F: Copy-neutral LOH (e.g., uniparental disomy)

Allele-specific copy number:
Red = Allele with increased copy number
Blue = Allele with decreased or lost copy number

Supplementary Figure 3B

Pt 1

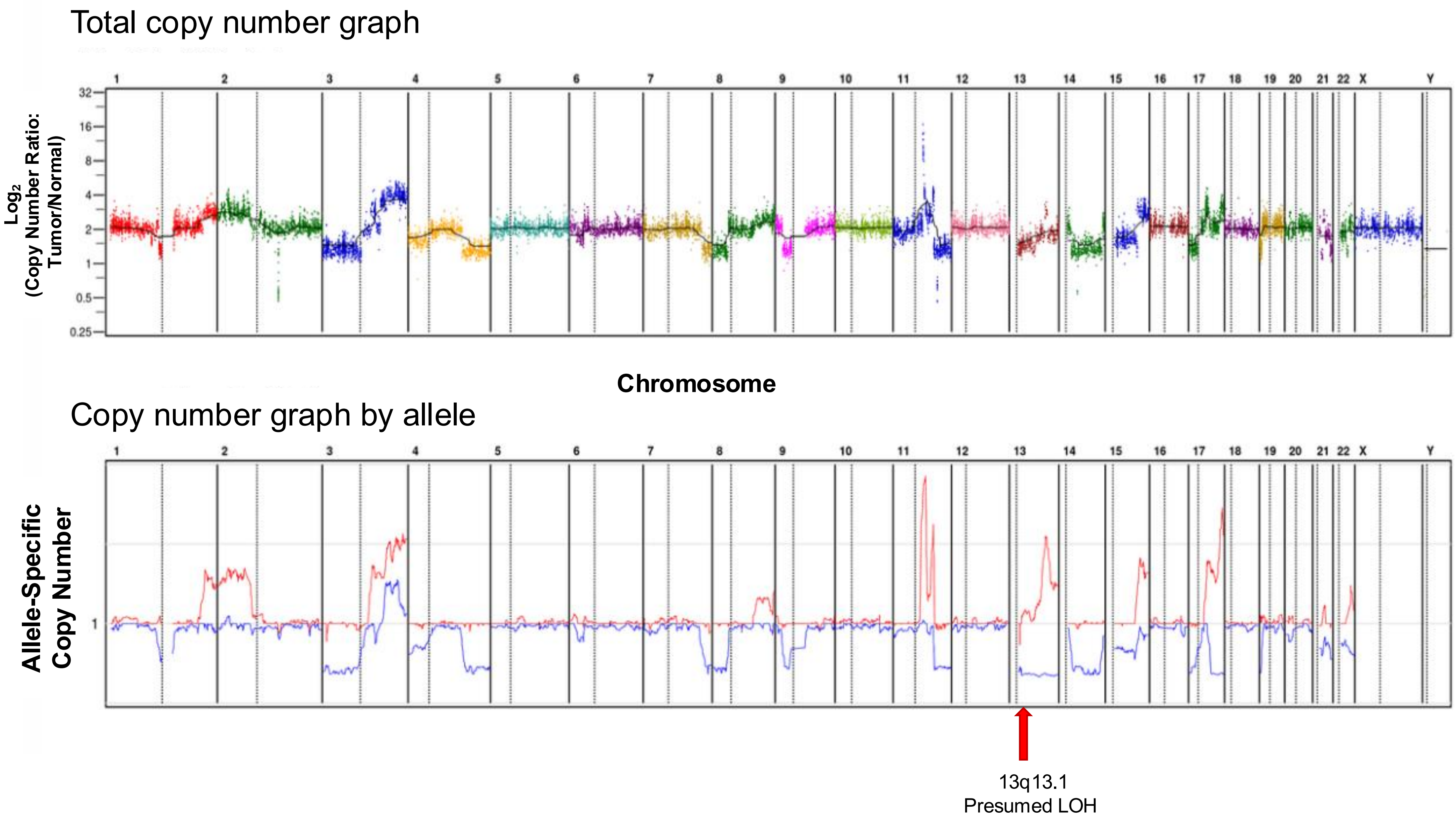

Supplementary Figure 3C

Pt 2

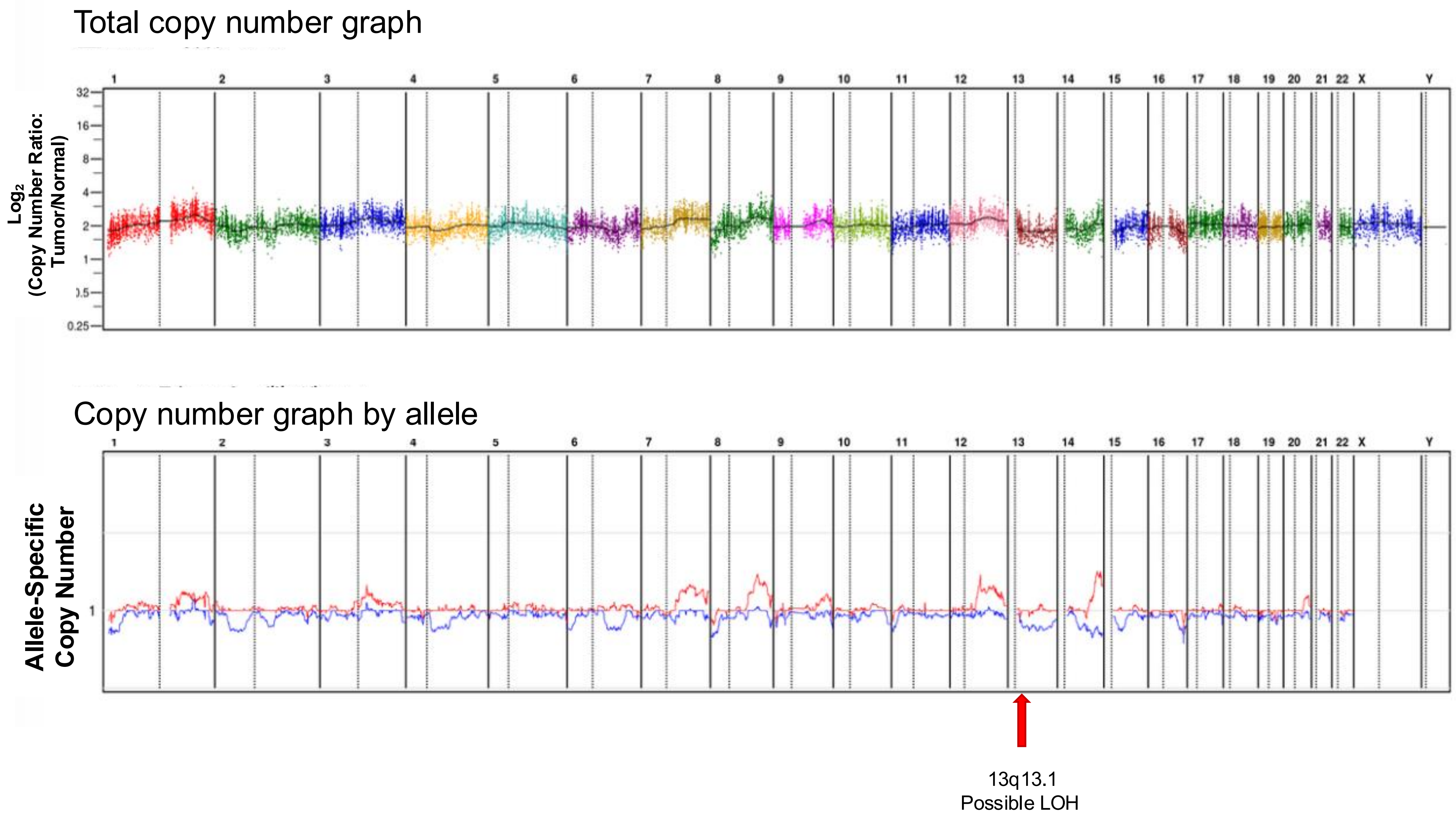

Pt 3

Supplementary Figure 3D

Total copy number graph

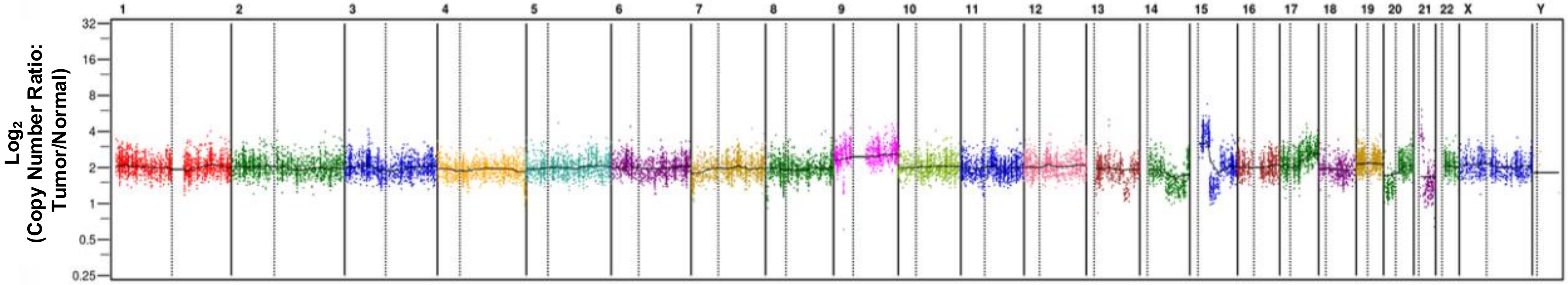

Copy number graph by allele

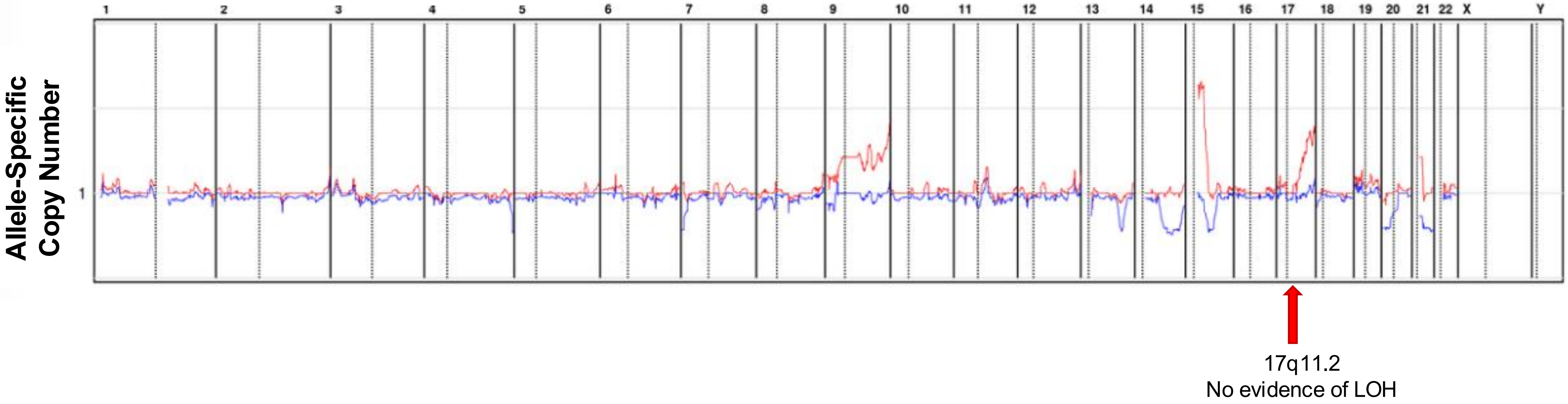

Supplementary Figure 3E

Pt 4

Total copy number graph

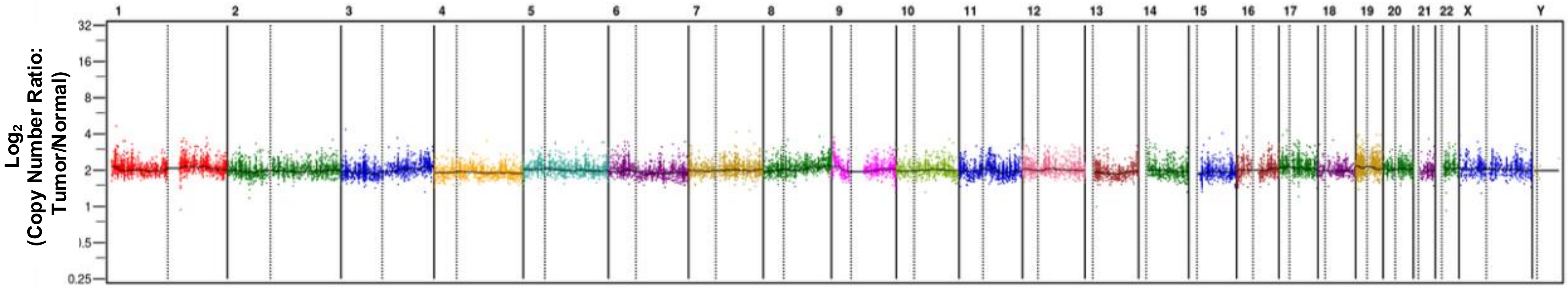

Copy number graph by allele

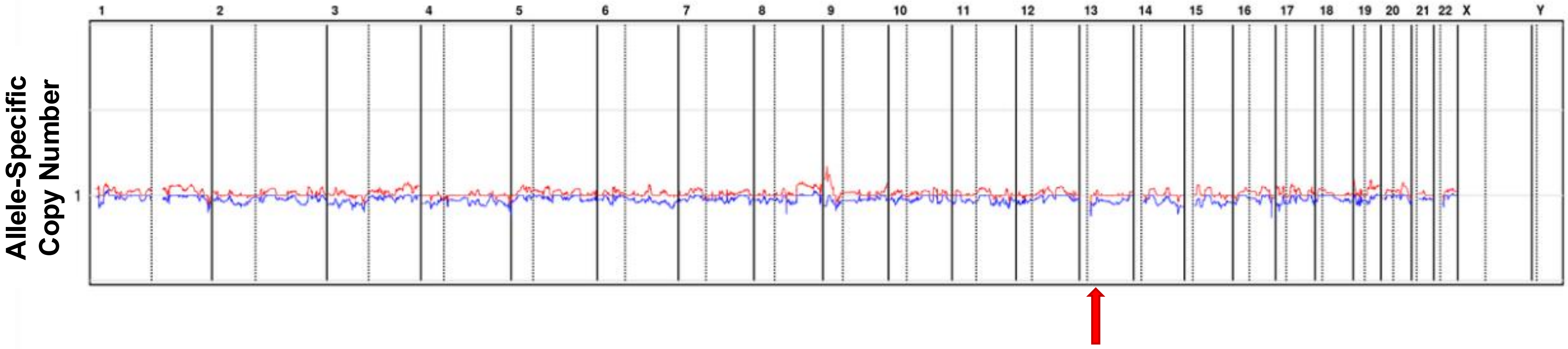

13q13.1
Inconclusive for LOH
