# Supplementary material for: Germline pathogenic variants detected by GenMineTOP: insight from a nationwide tumor/normal paired comprehensive genomic profiling test, in Japan
Source: J Hum Genet. 2025 Sep 9;71(1):1–11. doi: 10.1038/s10038-025-01389-z (PMC12689426; doi:10.1038/s10038-025-01389-z)
Supplement: Supplementary file 4 — Supplementary Table 1. Disclosure Recommendation List of Germline Findings in Cancer Genomic Profiling Tests (Kosugi Group List ver.4.2) [file 10038_2025_1389_MOESM4_ESM.pdf]

Supplementary Table 1. Comprehensive Tumor Genomic Profiling:
List of secondary findings to be disclosed to patients by the level of recommendation
(Kosugi Group List ver.4.2)

**Potentially Actionable SF Gene List**			**Level of recommendation for disclosure from the medical perspective (actionability) when pathological variants are confirmed in germline (Note 1)**	**Criteria for determining whether germline confirmatory testing should be performed when PGPV* is detected in the T-only Panel, and recommendation level (Note 2)**
**Gene**	**Major Phentype**	**Remarks**		
APC	FAP		AAA	○age<30
ATM	Cancer Predisposition Synd		AA	◎
BAP1	BAP1 Tumor Predisposition Synd		AA	○
BARD1	Cancer Predisposition Synd		AA	◎
BMPR1A	Juvenile Polyposis		AAA	△
BRCA1	HBOC		AAA	◎
BRCA2	HBOC		AAA	◎
BRIP1	Cancer Predisposition Synd		AA	◎
CDH1	HDGC		AA	○ (△Breast Ca)*
CDK4	Melanoma		B	□
CDKN2A	Melanoma/Pancreatic Ca		A	○age<30
CHEK2	Cancer Predisposition Synd		A	◎
DICER1	DICER synd		A	○
EPCAM	Lynch	Deletion	AA	□
FH	Hereditary Leiomyomatosis and Renal Cell Cancer (HLRCC)		AA	◎
FLCN	Birt-Hogg-Dubé Syndrome (BHD)		AA	◎
HNF1A	MODY3	Non-tumor	A	□
MAX	HPPS		AA	△
MEN1	MEN1		AAA	○
MET	Hereditary Papillary Renal Cancer (HPRC)		AA	□
MLH1	Lynch		AAA	◎
MSH2	Lynch		AAA	◎
MSH6	Lynch		AAA	◎
MUTYH	MAP	Biallelic	AA	◎
NF1	NF1		AA	○age<30 & Associated tumor type#
NF2	NF2		AA	△
NTHL1	FAP	Biallelic	B	□
PALB2	Cancer Predisposition Synd		AAA	◎

PMS2	Lynch		AAA	◎
POLD1	Polymerase Proofreading-Associated Polyposis (PPAP)		AA	◎
POLE	Polymerase Proofreading-Associated Polyposis (PPAP)		AA	◎
POT1	Malignant Melanoma		B	□
PTCH1	Gorlin Synd		B	□
PTEN	PTEN Hamartoma		AAA	△
RAD51C	Cancer Predisposition Synd		AA	◎
RAD51D	Cancer Predisposition Synd		AA	◎
RB1	Retinoblastoma		AAA	○age<30
RET	MEN2		AAA	◎
SDHA	HPPS		A	◎
SDHAF2	HPPS		AA	◎
SDHB	HPPS		AA	◎
SDHC	HPPS		AA	◎
SDHD	HPPS		AA	◎
SMAD3	Loeys-Dietz	non-tumor	A	□
SMAD4	Juvenile Polyposis		AAA	△
SMARCA4	Rhabdoid Tumor Predisposition Synd		B	◎age<30
SMARCB1	Rhabdoid Tumor Predisposition Synd		A	□
STK11	Peutz-Jeghers		AAA	△
SUFU	Gorlin Synd		B	□
TGFBR1	Loeys-Dietz	non-tumor	A	□
TGFBR2	Loeys-Dietz	non-tumor	A	△
TMEM127	Pheochromocytoma		AA	◎
TP53	Li-Fraumeni		AAA	○age<30 &Associated tumor type##
TSC1	Tuberous Sclerosis Complex		AA	△
TSC2	Tuberous Sclerosis Complex		AA	○
VHL	VHL		AAA	◎(△Renal tumor)**
WT1	WT1-related Wilms		AA	△

Note 1: Level of recommendation for disclosure from the medical perspective (actionability) when pathological variants are confirmed in germline

Grade: Explanation

AAA: Medical practice guidelines for pathological variant carriers are available in Japan or are equivalent to such guidelines.

AA: Hereditary tumor-causing genes in the ACMGSFv3 (73 genes)
Genes listed in the NCCN guidelines for which surveillance is recommended for disclosure.

A: Genes listed in the NCCN guidelines recommended for disclosure inconsistently in major articles
Other genes strongly recommended for disclosure consistently in major articles
Causative genes other than hereditary tumor-causing genes in the ACMGSFv3 (73genes)

B: Genes recommended for disclosure only in some articles

Note 2: Criteria for determining whether germline confirmatory testing should be performed when PGPV* is detected in the T-only Panel, and recommendation level

Grade: Explanation

◎**:** Confirmatory test should be performed, in principle, as the germline conversion rate is high (generally ≥50%)

O: Confirmatory test should be performed, if possible, as the germline conversion rate is somewhat high (approximately 10–50%)

□**:** Confirmatory test should be performed only in the presence of associated phenotypes, as data on the germline conversion rate is insufficient and other related limitations exist.

△**:** Confirmatory test should be performed, only in the presence of associated phenotypes, as the germline conversion rate is low (generally ≤5%)

Description of tumor name: Confirmatory test should be performed when the sample tumor (primary site) is described

Description of age: Confirmatory test should be performed when the patient's age meets the described conditions

＊: In breast cancer, confirmatory testing is recommended for cases with young-onset, lobular carcinoma, or diffuse gastric cancer phenotypes.

＊＊: In the case of renal tumor, confirmatory test should be performed in the presence ofphenotypes of juvenile or other VHL disease

# Associated Tumor Types:

Breast Cancer, CNS Cancer, Glioma, Nerve Sheath Tumor, Peripheral Nervous System Tumors, Pheochromocytoma-Paraganglioma (PHEO-PGL)

# # Additional Associated Tumor Types:

Adrenocortical Carcinoma, Bone Cancer, Breast Cancer, CNS Cancer, Colorectal Cancer, Embryonal Tumor, Gestational Trophoblastic Disease, Glioma, Soft Tissue Sarcoma, Wilms Tumor

Microsatellite instability-high (MSI-H) and other hypermutated samples should undergo confirmatory testing based on the same germline conversion rate grading criteria as non-hypermutated samples.

* Presumed Germline Pathogenic Variant refers to a pathological variant of a possible germline origin detected using Tonly panel.
If T-only panel is used, the decision shall be made regarding whether to disclose the findings based on the level of recommendation for disclosure as well as on the decision to perform a confirmatory germline test for the relevant PGPV.
Example 1) PGPV detected in TP53: Although the recommendation level was AAA, the patient was 65 years old and the tumor was not LFS-related; therefore, the expert panel determined that the significance of suggesting a confirmatory germline test is low and decided "not to disclose" the relevant PGPV.
Example 2) PGPV detected in RAD51D: The institution considered that findings with AA-level recommendation should be disclosed. Based on the criteria for confirmatory germline testing for the relevant PGPV (◎), the expert panel decided to "disclose" the relevant PGPV so as to suggest a confirmatory test to the patient.
Example 3) PGPV detected in PTEN: Although the recommendation level was AAA, the grade was (△) on the criteria scale for confirmatory germline testing; therefore, phenotypic evaluation was requested through the genetic medicinesection.
As a result, the expert panel decided "not to disclose" the relevalnt PGPV because the phenotype of PTEN hamartoma syndrome was not found.
