# Supplementary material for: Germline pathogenic variants detected by GenMineTOP: insight from a nationwide tumor/normal paired comprehensive genomic profiling test, in Japan
Source: J Hum Genet. 2025 Sep 9;71(1):1–11. doi: 10.1038/s10038-025-01389-z (PMC12689426; doi:10.1038/s10038-025-01389-z)
Supplement: Supplementary file 5 — Supplementary Table 2. Classification of On-Tumor and Off-Tumor Cancer Types by Gene [file 10038_2025_1389_MOESM5_ESM.pdf]

Supplementary Table 2.
Classification of On-Tumor and Off-Tumor Cancer Types by Gene

Gene	Associated tumour types (on-tumour)	Refarence
APC	・Colorectal cancer<br>・Duodenal or periampullary cancer<br>・Gastric cancer<br>・Small bowel cancer<br>・Intra-abdominal desmoid tumors<br>・Thyroid cancer<br>・Hepatoblastoma<br>・CNS tumor	Genetic/Familial High-Risk Assessment: Colorectal, Endometrial, and Gastric. Version 4.2024, 04/02/25 © 2025 National Comprehensive Cancer Network® (NCCN®)
ATM	・Breast cancer<br>・Epithelial ovarian cancer<br>・Pancreatic cancer<br>・Prostate cancer	Genetic/Familial High-Risk Assessment: Breast, Ovarian, Pancreatic, and Prostate. Version 3.2025, 03/06/25 © 2025 National Comprehensive Cancer Network® (NCCN®)<br>Genetic/Familial High-Risk Assessment: Colorectal, Endometrial, and Gastric. Version 4.2024, 04/02/25 © 2025 National Comprehensive Cancer Network® (NCCN®)
BRCA1	・Breast cancer<br>・Epithelial ovarian cancer<br>・Pancreatic cancer<br>・Prostate cancer	Genetic/Familial High-Risk Assessment: Breast, Ovarian, Pancreatic, and Prostate. Version 3.2025, 03/06/25 © 2025 National Comprehensive Cancer Network® (NCCN®)
BRCA2	・Breast cancer<br>・Epithelial ovarian cancer<br>・Pancreatic cancer<br>・Prostate cancer<br>・Melanoma	Genetic/Familial High-Risk Assessment: Breast, Ovarian, Pancreatic, and Prostate. Version 3.2025, 03/06/25 © 2025 National Comprehensive Cancer Network® (NCCN®)
BRIP1	・Epithelial ovarian cancer	Genetic/Familial High-Risk Assessment: Breast, Ovarian, Pancreatic, and Prostate. Version 3.2025, 03/06/25 © 2025 National Comprehensive Cancer Network® (NCCN®)
CDH1	・Gastric cancer (Diffuse or signet ring cell carcinomab)<br>・Breast cancer (Lobular)	Genetic/Familial High-Risk Assessment: Colorectal, Endometrial, and Gastric. Version 4.2024, 04/02/25 © 2025 National Comprehensive Cancer Network® (NCCN®)

CHEK2	・Breast cancer<br>・Prostate cancer	Genetic/Familial High-Risk Assessment: Breast, Ovarian, Pancreatic, and Prostate. Version 3.2025, 03/06/25 © 2025 National Comprehensive Cancer Network® (NCCN®)
MEN1	・Parathyroid adenoma/hyperplasia<br>・PanNETs (functioning) or duodenal NETs<br>・Pituitary adenomas (30%–40%)<br>・Gastric carcinoids (7%–35%)<br>・Lung/thymic carcinoids (<8%)<br>・Adrenal adenomas (27%–36%)	Neuroendocrine and Adrenal Tumors Version 1.2025, 03/27/2025 © 2025 National Comprehensive Cancer Network® (NCCN®)
MLH1	・Colorectal cancer<br>・Endometrial cancer<br>・Ovarian cancer<br>・Renal pelvis and/or ureter cancer<br>・Bladder cancer<br>・Gastric cancer<br>・Small bowel cancer<br>・Pancreatic cancer<br>・Biliary tract cancer<br>・Prostate cancer<br>・Brain tumor	Genetic/Familial High-Risk Assessment: Colorectal, Endometrial, and Gastric. Version 4.2024, 04/02/25 © 2025 National Comprehensive Cancer Network® (NCCN®)
MSH2	・Colorectal cancer<br>・Endometrial cancer<br>・Ovarian cancer<br>・Renal pelvis and/or ureter cancer<br>・Bladder cancer<br>・Gastric cancer<br>・Small bowel cancer<br>・Pancreatic cancer<br>・Biliary tract cancer<br>・Prostate cancer<br>・Brain tumor<br>・Skin（*MSH2* c.942+3A>T variant carriers）	Genetic/Familial High-Risk Assessment: Colorectal, Endometrial, and Gastric. Version 4.2024, 04/02/25 © 2025 National Comprehensive Cancer Network® (NCCN®)

MSH6	・Colorectal cancer<br>・Endometrial cancer<br>・Ovarian cancer<br>・Renal pelvis and/or ureter cancer<br>・Bladder cancer<br>・Gastric cancer<br>・Small bowel cancer<br>・Pancreatic cancer<br>・Biliary tract cancer<br>・Prostate cancer<br>・Brain tumor	Genetic/Familial High-Risk Assessment: Colorectal, Endometrial, and Gastric. Version 4.2024, 04/02/25 © 2025 National Comprehensive Cancer Network® (NCCN®)
NF1	・Breast cancer<br>・Malignant peripheral nerve sheath tumors<br>・gastrointestinal stromal tumors (GIST)	Genetic/Familial High-Risk Assessment: Breast, Ovarian, Pancreatic, and Prostate. Version 3.2025, 03/06/25 © 2025 National Comprehensive Cancer Network® (NCCN®)
NF2	・Bilateral vestibular schwannomas<br>・Other intracranial and spinal schwannomas<br>・Meningiomas<br>・Ependymomas<br>・low-grade astrocytomas	NCBI GeneReviews:<br>NF2-Related Schwannomatosis<br>https://www.ncbi.nlm.nih.gov/books/NBK1201/
PALB2	・Breast cancer<br>・Epithelial ovarian cancer<br>・Pancreatic cancer	Genetic/Familial High-Risk Assessment: Breast, Ovarian, Pancreatic, and Prostate. Version 3.2025, 03/06/25 © 2025 National Comprehensive Cancer Network® (NCCN®)
PTEN	・Breast cancer<br>・Endometrial cancer<br>・Colon cancer<br>・Kidney cancer<br>・Neurologic<br>・Skin<br>・Thyroid	Genetic/Familial High-Risk Assessment: Breast, Ovarian, Pancreatic, and Prostate. Version 3.2025, 03/06/25 © 2025 National Comprehensive Cancer Network® (NCCN®)<br>Genetic/Familial High-Risk Assessment: Colorectal, Endometrial, and Gastric. Version 4.2024, 04/02/25 © 2025 National Comprehensive Cancer Network® (NCCN®)
RAD51C	・Breast cancer<br>・Epithelial ovarian cancer	Genetic/Familial High-Risk Assessment: Breast, Ovarian, Pancreatic, and Prostate. Version 3.2025, 03/06/25 © 2025 National Comprehensive Cancer Network® (NCCN®)

RAD51D	・Breast cancer<br>・Epithelial ovarian cancer	Genetic/Familial High-Risk Assessment: Breast, Ovarian, Pancreatic, and Prostate. Version 3.2025, 03/06/25 © 2025 National Comprehensive Cancer Network® (NCCN®)
RB1	・Retinoblastoma<br>・Pineoblastoma<br>・Osteosarcoma<br>・Soft tissue sarcoma (mostly leiomyosarcoma and rhabdomyosarcoma)<br>・Melanoma	NCBI GeneReviews: Retinoblastoma https://www.ncbi.nlm.nih.gov/books/NBK1452/
SDHA	・Pheochromocytoma<br>・Paraganglioma<br>・GI stromal tumor (GIST)<br>・Renal cell cancer	Neuroendocrine and Adrenal Tumors Version 1.2025, 03/27/2025 © 2025 National Comprehensive Cancer Network® (NCCN®)
SDHB	・Pheochromocytoma<br>・Paraganglioma<br>・GI stromal tumor (GIST)<br>・Renal cell cancer	Neuroendocrine and Adrenal Tumors Version 1.2025, 03/27/2025 © 2025 National Comprehensive Cancer Network® (NCCN®)
TP53	・Soft tissue sarcoma<br>・Osteosarcoma<br>・CNS tumor<br>・ACC<br>・Breast cancer<br>・Pancreatic cancer<br>・Melanoma<br>・Colorectal cancer<br>・Gastric cancer<br>・Prostate cancer	Genetic/Familial High-Risk Assessment: Breast, Ovarian, Pancreatic, and Prostate. Version 3.2025, 03/06/25 © 2025 National Comprehensive Cancer Network® (NCCN®)<br>Genetic/Familial High-Risk Assessment: Colorectal, Endometrial, and Gastric. Version 4.2024, 04/02/25 © 2025 National Comprehensive Cancer Network® (NCCN®)
