# Supplementary material for: Germline pathogenic variants detected by GenMineTOP: insight from a nationwide tumor/normal paired comprehensive genomic profiling test, in Japan
Source: J Hum Genet. 2025 Sep 9;71(1):1–11. doi: 10.1038/s10038-025-01389-z (PMC12689426; doi:10.1038/s10038-025-01389-z)
Supplement: Supplementary file 10 — Tables Legend [file 10038_2025_1389_MOESM10_ESM.docx]

**Supplementary Table 1. Comprehensive Tumor Genomic Profiling: List of secondary findings to be disclosed to patients by the level of recommendation (Kosugi Group List ver.4.2)**

This table lists 53 genes deemed clinically actionable in Japan when GPVs are identified through CGP or other genetic analyses. The selection is based on ACMG SF v3.2 and is referred to as the "Kosugi Group List." The table includes gene names, major phenotypes, evidence levels, and criteria for inclusion in tumor-only panels.

**Supplementary Table 2. Classification of On-Tumor and Off-Tumor Cancer Types by Gene**

Classification of “on-tumor” and “off-tumor” cancer types for each gene. Based on NCCN Guidelines® (2024–2025) and relevant literature. Used to assess tumor–gene relevance in this study.

**Supplementary Table 3. Clinical Characteristics of Patients Who Underwent Testing with the GenMineTOP**

This table summarizes patient demographics, including age distribution, gender ratio, and median age, for a total of 1,356 patients analyzed in the study.

**Supplementary Table 4. Details of 73 Patients with GPVs in GenMineTOP**

This table lists individual patients with GPVs, including their cancer type, on-tumor or off-tumor status, sex, age, TMB value, gene, variant, variant allele frequency, and the variant classification according to the ACMG/AMP 2015 guidelines. If a patient has two variants, additional variant information is also included.

**Supplementary Table 5. Recommendation Grades of GPVs Detected in GenMineTOP Based on the Kosugi Group List**

This table presents the recommendation grades (B to AAA) of GPVs detected in GenMineTOP according to the Kosugi Group List, along with their coverage by the NCC OncoPanel and the number of patients in this study.

**Supplementary Table 6. Details of GPVs and Genetic Counseling for 5 Patients in Our Institution**

This table provides detailed information on five patients with germline pathogenic variants, their clinical characteristics, genetic counseling status, presumed loss of heterozygosity, and additional genetic findings.

**Supplementary Figure 1. Kosugi Group List Disclosure Genes and Target Genes in Each Panel**

(A) The 53 genes designated for disclosure in the Kosugi Group List (Ver 4.2), along with the target genes analyzed in GenMineTOP (40 genes, until October 9th, 2024) and in NOP (124 genes).

(B) The 53 genes designated for disclosure in the Kosugi Group List (Ver 4.2), along with the target genes analyzed in GenMineTOP (59 genes, since October 10th, 2024) and in NOP (124 genes).

**Supplementary Figure 2.**

A table with GPVs as row headers and cancer types as column headers. Patient age is categorized into three groups and color-coded: ≥65 years old (green), 40–64 years old (orange), and 0–39 years old (purple). HR-related genes are highlighted in yellow.

**Supplementary Figure 3.**

(A) How to Interpret Copy Number and Allele-Specific Copy Number Graphs

(B–E) Genome-wide copy number and allele-specific graphs for Pt 1–4. Arrows indicate regions with allelic imbalance suggestive of LOH, based on divergence of major (red) and minor (blue) allele copy number. Pt 3 exhibited LOH at 17q11.2, where the minor allele was lost and the major allele was retained, supporting a hemizygous deletion in the region. Pt 4 did not show evidence of LOH at 13q13.1, as both red and blue allele-specific copy number lines were retained within the expected levels.
